# Phylogeography of the *Chydorus sphaericus* Group (Cladocera: Chydoridae) in the Northern Palearctic

**DOI:** 10.1371/journal.pone.0168711

**Published:** 2016-12-19

**Authors:** Alexey A. Kotov, Dmitry P. Karabanov, Eugeniya I. Bekker, Tatiana V. Neretina, Derek J. Taylor

**Affiliations:** 1 Laboratory of Aquatic Ecology and Invasions, A. N. Severtsov Institute of Ecology and Evolution of Russian Academy of Sciences, Moscow, Russia; 2 Laboratory of Fish Ecology, I. D. Papanin Institute for Biology of Inland Waters of Russian Academy of Sciences, Borok, Yaroslavl Area, Russia; 3 White Sea Biological Station, Biological Faculty, M.V. Lomonosov Moscow State University, Moscow, Russia; 4 Department of Biological Sciences, The State University of New York at Buffalo, Buffalo, United States of America; University of Otago, NEW ZEALAND

## Abstract

The biodiversity and the biogeography are still poorly understood for freshwater invertebrates. The crustacean *Chydorus sphaericus-brevilabris* complex (Cladocera: Chydoridae) is composed of species that are important components of Holarctic freshwater food webs. Recent morphological and genetic study of the complex has indicated a substantial species diversity in the northern hemisphere. However, we know little of the geographic boundaries of these novel lineages. Moreover, a large section of the Palearctic remains unexamined at the genetic level. Here we attempt to address the biodiversity knowledge gap for the *Chydorus sphaericus* group in the central Palearctic and assess its diversity and biogeographic boundaries. We sequenced nuclear (*ITS-2*) and mitochondrial (*COI*) gene regions of *Chydorus* specimens across the Palearctic and compared them with already available Holarctic sequences. We detected six main clades in the *C*. *sphaericus* group in the Palearctic, of which two of the groups are novel. Three of the more divergent clades are geographically widespread. The central portion of Eurasia (the Yenisey River basin) appears to be a narrow zone of secondary contact for phylogroups that expanded from European and Beringian refugia. As such, the previously unsampled central Palearctic represents an important region for understanding the evolutionary consequences of Pleistocene climatic oscillations on the *Chydorus sphaericus* group.

## Introduction

Cladocerans (Branchiopoda) are well-studied crustaceans in aquatic biology [1]. The best studied cladocerans belong to the planktonic genus *Daphnia* [2–4], but a few recent studies have investigated non-planktonic genera [5–10]. Planktonic and littoral cladocerans often differ in their resting egg structure and dispersal strategy. Females of the Order Anomopoda Sars place their resting eggs in ephippia–modified exuvia. There is evidence that the ephippia of various pelagic cladocerans are adapted for easy dispersal from one water body to another by waterfowl or by some abiotic vectors [11–13]. However, the ephippia of littoral anomopods are heavy and appear adapted to be kept in the water body of the parents [14–15].

The best known genus of the littoral cladocerans is *Chydorus* Leach, the type genus of the family Chydoridae Dybowski & Grochowski. This family is the most species-rich among the cladocerans. The first taxon of this genus was described by O. F. Müller [16] as *Lynceus sphaericus* O.F. Müller, 1776. Leach [17] later established the genus *Chydorus* Leach, 1816 for a single species *C*. *sphaericus*. Since the revision of Leach, many authors noted the presence of the genus in different regions of the world, and many new taxa were described [18–27]. It soon became apparent that taxa of this genus are among most common cladoceran species in many regions of the world. Moreover, *Chydorus* was detected in a very wide range of water bodies, from minute puddles to large lakes and rivers, and from oligotrophic alpine lakes to hypertrophic lowland reservoirs [28–29]. *Chydorus sphaericus* was recorded innumerable times in studies of freshwater from the 19th-21st centuries.

Smirnov [30] provided a major revision of the family Chydoridae. He noted the existence of a great number of taxa being synonyms of earlier described species in some groups, including *Chydorus*. Twenty three species of *Chydorus* were found to be valid, while some species were regarded as having several subspecies [30]. But the diversity of the genus remained poorly understood at the global level. This made adequate biogeographic conclusions concerning *Chydorus* difficult.

Frey [31–38] then started another spate of taxonomic revisions of *Chydorus*, and found that many "taxa" earlier regarded as cosmopolitan are represented by groups of species with relatively narrow distributions–single continents or smaller regions [39–40]. Efforts to revise the taxonomy of some species groups of *Chydorus* based on morphological characters were continued by a few authors [41–50]. Valuable descriptions of some species were also presented in regional publications [47, 51]. Also, several groups of the *Chydorus*-like cladocerans were assigned to separate genera [34, 52]. As a result, many taxonomic problems were resolved and biogeographic boundaries started to become apparent.

Another wave of advances occurred in cladoceran systematic biology with the application of genetic methods. In addition to an external method to evaluate the correctness of the separation and grouping of some taxa, we now have a powerful tool to understand their evolutionary history, reconstructing genealogical relationships within each group based on molecular markers. Such works for chydorids are only beginning to appear [6–7].

The most comprehensive study of chydorid taxonomy, biogeography and evolution based on molecular markers was conducted by Belyaeva & Taylor [7] for the Holarctic *Chydorus sphaericus-brevilabris* complex. The authors found that there are at least seven taxa in the *C*. *sphaericus* group in the Holarctic, including *C*. *biovatus*. In agreement with the opinion of Frey [31, 37], *C*. *brevilabris* group was found to be a sister group to the *sphaericus*-group based on molecular methods. But Belyaeva & Taylor [7] lacked material from a large geographic area, the Eastern Palearctic (only a few populations from the Eastern coast of Asia were represented). Due to this knowledge gap, it is presently difficult to make detailed conclusions about the biogeography, diversity and phylogeography of Palearctic *Chydorus*.

Other recent investigations on the diversity of the genus *Chydorus* resulted from the DNA barcoding of the cladocerans in the New World [53–57]. These data were largely composed of samples beyond the *Chydorus sphaericus*-group.

Our study aims to address the taxonomic and biogeographic knowledge gap for the *Chydorus sphaericus* species group in the Eastern Palearctic. We combine original DNA sequences of the mitochondrial cytochrome c oxidase (*COI)* and nuclear rDNA internal transcribed spacer (*ITS-2*) genes with those deposited in Genbank to assess phylogeography, biogeographic boundaries and taxonomic representation of this group in the Palearctic.

## Materials and Methods

### Collection of samples

Field collection in Russia was carried out by our team or by our colleagues under the fulfillment of the governmental program "Ecology and biodiversity of aquatic ecosystems and invasions of alien species" (№ 0109-2014-0008), or during engineering ecology surveys with governmental permission to collect such samples from public property. Sampling in the natural reserves of Russia was conducted with special permission of their Directors (A.L. Strelnikov, Komandorsky State Natural Reserve; T. I. Shpilenok, Kronotsky Biosphere Reserve; Yu.P. Suschitsky, State Khankaisky Biosphere Reserve). All collected samples were listed in special reports to the administration of the reserves. Verbal permissions to collect in private farm ponds were obtained from local owners. Field collections in South Korea were carried out as part of the fulfillment of the program from the National Institute of Biological Resources with governmental permission to collect on public property. Samples from Norway were provided by I. Diemantovica, under permission to collect as a member of a Norwegian government institute. The field studies and collections did not involve endangered or protected species.

Specimens were collected by plankton nets with a diameter of 0.20–0.4 m and a mesh size of 30–50 μm, or rectangular dip nets of the same mesh size but a width of 0.2–0.3 m. Collections were preserved in 90–96% alcohol. Before the start of genetic studies, ten specimens of *Chydorus* from each locality were preliminarily identified by morphological characters [7, 50]. Only members of the *C*. *sphaericus* group were incorporated into this study. One to five specimens from each of 22 localities through whole Palaearctic were studied here.

### DNA sequencing

Genomic DNA was extracted using a Wizard Genomic DNA Purification Kit (Promega Corporation, Madison, WI, USA) according to the manufacturer’s instructions. The polymerase chain reaction (PCR) was used to amplify a 461 bp fragment of the 5’ region of the mitochondrial cytochrome c oxidase subunit I gene (*COI*) using the primers Chy-f and Chy-r [7]. A subsample of 14 individuals was sequenced for the nuclear fragment, which included the complete sequence of internal transcribed spacer 2 (*ITS-2*) as well as small partial sequences of 5.8S and 28S ribosomal RNA genes, using the primers 5.8SF [58] and D2r [59]. In the text below we refer to the nuclear fragment as *ITS-2* for convenience. The 25 μl PCR reaction consisted of 2 μl of genomic DNA, 8.5 μl of double-distilled H_2_O, 1 μl of each primer (10 mM) and 5 μl PCR 5x Taq ScreenMix-HS (Evrogen, Moscow, Russia). The PCR conditions for the *COI* amplification were 1 cycle of 5 min at 95°C, 40 cycles of 40 s at 94°C (denaturation), 30 s at 50°C (annealing) and 90 s at 72°C (extension), followed by 1 cycle of 7 min at 72°C. The PCR conditions for the amplification of *ITS-2* were the same, but the annealing temperature was 60°C [7]. PCR products were purified by precipitation in ethanol and sequenced bi-directionally on an ABI 3730 DNA Analyzer with ABI PRISM BigDye Terminator v. 3.1 sequencing kit (Applied Biosystems, USA). A single consensus sequence was assembled using the forward and reverse sequences using CodonCode Aligner v. 6.0.2 (CodonCode Corp, USA). DNA sequences were submitted to the NCBI GenBank database (accession Nos. KX431583-KX431669 for *COI* and KX 448799-KX448812 for *ITS-2* sequences).

### Phylogenetic analyses

The initial authenticity of the sequences was verified by BLAST (blastn) comparisons. The mitochondrial sequences were translated using the invertebrate mitochondrial genetic code to verify the open reading frame. The original sequences from the present study (S1 Table) and known sequences from GenBank (S2 Table) assigned to the *C*. *sphaericus-brevilabris* complex were submitted for alignment. The sequences KC616878, KC617532, KC617533, KC617535 by Prosser et al. [58] were excluded from our analysis as they apparently do not belong to the genus *Chydorus*. Sequences were edited and assembled in the UGENE v. 1.23 package [60]. The DNA sequences were first automatically aligned using the Clustal Omega algorithm [61] using default options and then manually edited. The following population genetics values were estimated: the number of polymorphic sites (S), number of haplotypes (h), haplotype diversity (Hd), nucleotide diversity (Pi) and average number of nucleotide differences (k). All calculations were performed using DnaSP v. 5.1 [62] and MEGA v.7 [63].

For tests of neutrality, we calculated Tajima’s D [64] and Fu’s Fs [65] using Arlequin v. 3.5 [66] with 10000 permutations. Mismatch distribution was constructed for each geographic population to test a model of exponential population growth [67]. A goodness of fit test was performed to test the validity of the sudden expansion model, using a parametric bootstrap approach based on the sum of square deviations (SSD) between the observed and simulated mismatch distributions, with P-values calculated as the proportion of simulations producing an SSD larger than or equal to the observed SSD. The demographic parameter Tau was estimated using a generalized nonlinear least square approach, and the confidence interval of this parameter was computed using a parametric bootstrap with 10000 replicates in Arlequin 3.5.

The best-fitting models of nucleotide substitution were selected in jModelTest 2.1.7 [68] based on the likelihood scores for 88 different models and the Akaike information criterion [69]. Within- and among-clade distances were calculated in MEGA7 using the Tamura 3-parameter model [70] with the shape parameter estimated by jModelTest for *COI* and the Kimura 2-parameter model [71] for *ITS-2*. Phylogenetic analyses were performed separately for each gene.

We used sequences from the chydorid genus *Pleuroxus* for outgroup rooting of the phylogenetic trees. The maximum likelihood (ML) phylogeny reconstruction was performed with MEGA7 and 1000 nonparametric bootstrap pseudoreplicates. Maximum parsimony (MP) analyses were performed in PAUP*4.0a149 [72]. Heuristic MP searches were done using equal weighting, 10 random sequence addition replicates and tree bisection and reconnection (TBR) branch swapping. Non-parametric bootstrapping was performed to assess the nodal support using 1000 pseudoreplicates for MP. Bayesian analyses (BI) were performed in MrBayes v.3.2.6 [73]. Four independent Markov chain Monte Carlo (MCMC) analyses were run simultaneously for 10 million generations and sampled every 1000 generations. The first 25% of the generations were discarded as the burn-in and a 50% majority rule consensus tree was calculated from the remaining trees. The mean genetic sequence divergence between major phylogroups was calculated in MEGA7 using models estimated by jModelTest with pairwise deletion of gaps.

The program Network 5 was employed to construct a median-joining haplotype network that is based on minimum spanning trees in which median vectors representing extinct central or unsampled haplotypes were added following the maximum-parsimony principle [74]. Central haplotypes were identified by their internal⁄central positions in the network, their high frequencies and the number of low-frequency haplotypes derived from them.

Both data sets were tested for recombination events using GARD software [75]. The model selection tool available on the web-server [76] was used to obtain the input nucleotide substitution model. The other settings were: general discrete model for rate variation with four rate classes.

## Results

### Sequence variation and alignment

**Fig 1:**
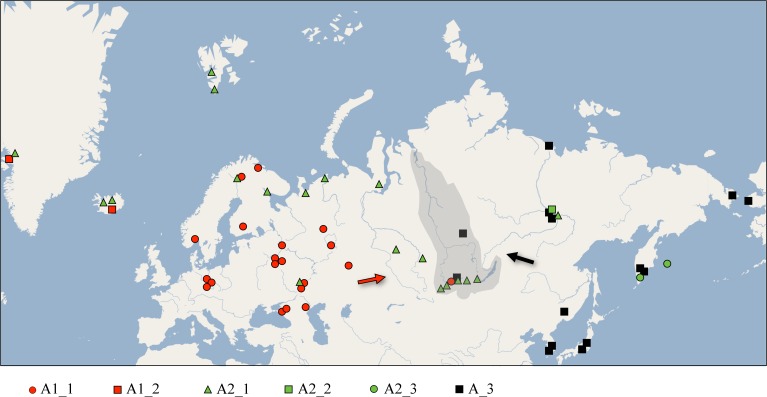
Eurasian sites from which diversity of the *Chydorus sphaericus* group has been analyzed. Geometric symbols for Eurasian taxa are corresponding to those in subsequent figures. Arrows represent possible dispersal routes from glaicial refugia. Gray shading–the Yenisey River basin, Siberia. The base map is from the open domain plain map available at https://marble.kde.org/.

The overall sequencing success rate for the mitochondrial gene was about 85%, negative results appeared due to improper sample preservation. Of 144 specimens from 22 localities (Fig 1) sequenced for *COI*, 64 unique haplotypes were detected. A summary of the diversity of this marker is presented in Tables 1 and 2, but only unique sequences were included in the phylogenetic analyses. The *COI* alignment for all ingroups and *Pleuroxus* was 461 bp long, unambiguous and contained no indels or reading frame disruptions. There were 87 variable and 75 parsimony informative sites, with 93 total substitutions. The average base composition was as follows: A–T = 61%. The observed A–T content around 60% is common for the *COI* region of chydorids [6]. The translated amino-acid alignment had 153 characters, of which 8 were variable and 6 parsimony informative. The overall transition/transversion bias was R = 6.554.

**Table 1:** Polymorphism metrics of the *COI* gene (mtDNA) in the clades of the A1+A2+A3 group from the *Chydorus sphaericus* group.

Clade	n	S	h	Hd	Pi	k	Tajima’s D		Fu’s Fs		Mismatch distributions	
							D	P	Fs	P	Tau	SSD (P_SSD_)
A1	49	24	21	0.876	0.007	3.40	-1.289	>0.10	-25.8	0	3.9	0.007 (0.74)
A2_1	21	25	16	0.971	0.012	5.64	-0.716	>0.10	-19.5	0	7.3	0.012 (0.47)
A2_2	18	12	10	0.922	0.005	2.34	-1.216	>0.10	-24.7	0	2.1	0.003 (0.31)
A2_3	1		1									
A3	45	19	16	0.824	0.006	2.72	-1.201	>0.10	-26.5	0	4.3	0.003 (0.91)
Total	134	87	64									

n—sample size, S—number of polymorphic sites, h—number of haplotypes, Hd—haplotype diversity, Pi—nucleotide diversity, k—average number of nucleotide differences and test results of Tajima’s D, Fu’s Fs and mismatch distributions.

**Table 2:** Between-group genetic distances of the “A”-groups of *Chydorus* for the *ITS-2* gene (Kimura 2-parameter) and *COI* gene (Tamura 3-parameter).

	A1	A2_1	A2_2	A2_3	A3
A1		0.012 / 0.006	0.011 / 0.019	0.009 / -	0.018 / 0.016
A2_1	0.055 / 0.073		0.009 / 0.020	0.008 / -	0.019 0.018
A2_2	0.050 / 0.308	0.032 / 0.318		0.009 / -	0.017 / 0.027
A2_3	0.037 / -	0.025 / -	0.034 / -		0.017 / -
A3	0.103 / 0.222	0.110 / 0.251	0.100 / 0.470	0.100 / -	

Distances were calculated assuming gamma distributed rates with a shape parameter estimated by jModelTest2. *ITS*-2 distances were calculated with pairwise deletion of gaps. Distances for *ITS-2* –above, for *COI*–below diagonal.

In *ITS-2* sequences, there were both very variable regions, and very conserved regions which displayed no variation. The variable regions were difficult to align and we routinely observed all individual sequences in the alignment. We controlled visually and edited manually all dubious substitutions, returning to the original electropherograms in each dubious case. Only 14 *ITS-2* sequences were homozygous in *ITS-2*. These gave good quality sequences with no or few ambiguities. The rest of the sequences were unreadable due to within-individual variation in the sequence and its length, resulting in ca. 60% of double electropherogram peaks. Such *ITS-2* heterozygotes were not associated with a particular clade, but rather they were present in each of the *COI*-defined clades. The final *ITS-2* alignment included only non-identical sequences from homozygous individuals, and main groups under consideration revealed by the *COI* phylogeny were represented. Outgroups were excluded as well, because their inclusion substantially decreased the quality of the alignment. The final *ITS-2* alignment had 708 substitutions and was 1076 characters long with 525 variable and 290 parsimony informative sites and contained numerous indels of 1–12 bp long. The average *ITS-2* base composition was: A–T = 47%. The overall transition/transversion bias was R = 0.621.

### *COI* phylogenetic tree

The best-fit model selected by jModeltest for the *COI* data set was Tamura 3-parameter (T92+G+I) with a gamma distribution and a parameter for the proportion of invariable sites. The T92 distances among *COI* ingroup taxa varied between 0% and 12%.

**Fig 2:**
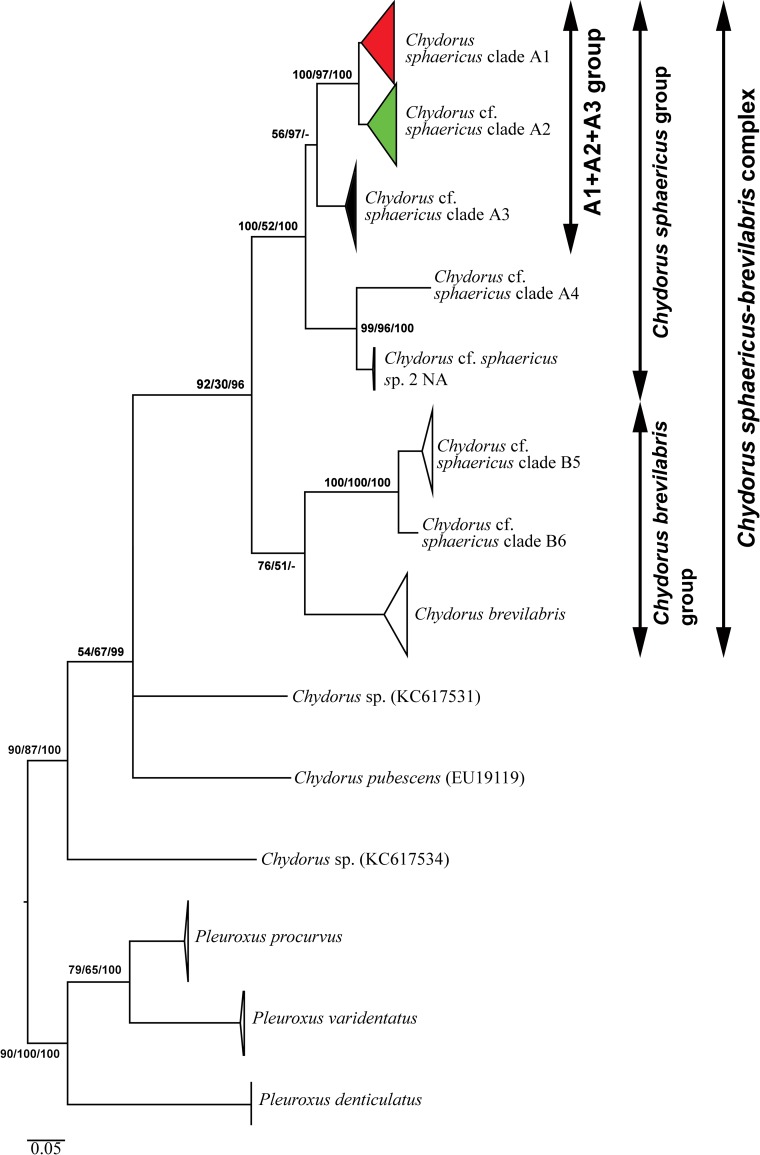
Maximum likelihood tree based on sequences of the mitochondrial cytochrome c oxidase subunit I (COI) gene representing the diversity among phylogroups of the genus *Chydorus*. The support values of individual nodes are based on: Maximum likelihood (ML)/ Maximum Parsimony (MP) / Bayesian Inference (BI). Colors for main phylogroups correspond to those in Fig 1.

Original sequences together with the GenBank sequences could be associated with 11 large clades of *Chydorus* in ML search (Fig 2). Trees constructed by different methods were congruent, but the clade support differed among analyses. In general, the clades having strong support in ML also had strong support in BI. Deep branches had moderate to strong support in ML and BI, which is not usual for trees based on *COI* alone. Among the apparent 11 phylogroups, eight belong to the *C*. *sphaericus-brevilabris* complex, while three clades (*C*. *pubescens* and two unassigned taxa from Genbank) are beyond this species complex. We named the main clades from Eurasia according to Belyaeva & Taylor [7]: A1–A3. The North American clades are also numbered according to existing names (A4, B5 and B6 from [7]; *Chydorus* cf. *sphaericus* sp. 2 NA from [57]). Within the *C*. *sphaericus-brevilabris* complex there are three main clades: (1) *Chydorus brevilabris* + *C*. cf. *sphaericus* clade B6 + *C*. cf. *sphaericus* clade B5 (with moderate support in ML and no support in BI) occurring exclusively in North America, these clades form *C*. *brevilabris* group; (2) *Chydorus* cf. *sphaericus* clade A4 + *C*. cf. *sphaericus* sp. 2 NA (with a strong support in all searches) also occurring only in North America; (3) *Chydorus sphaericus* clades A1 + A2 + A3 (with weak support in ML and BI, but strong support in MP) occurring predominantly in Eurasia (although the A3 clade was also found in northwestern North America, east to Manitoba). Two latter clades form *C*. *sphaericus* group, and our work is concentrated on the third group (A1 + A2 + A3) only.

**Fig 3:**
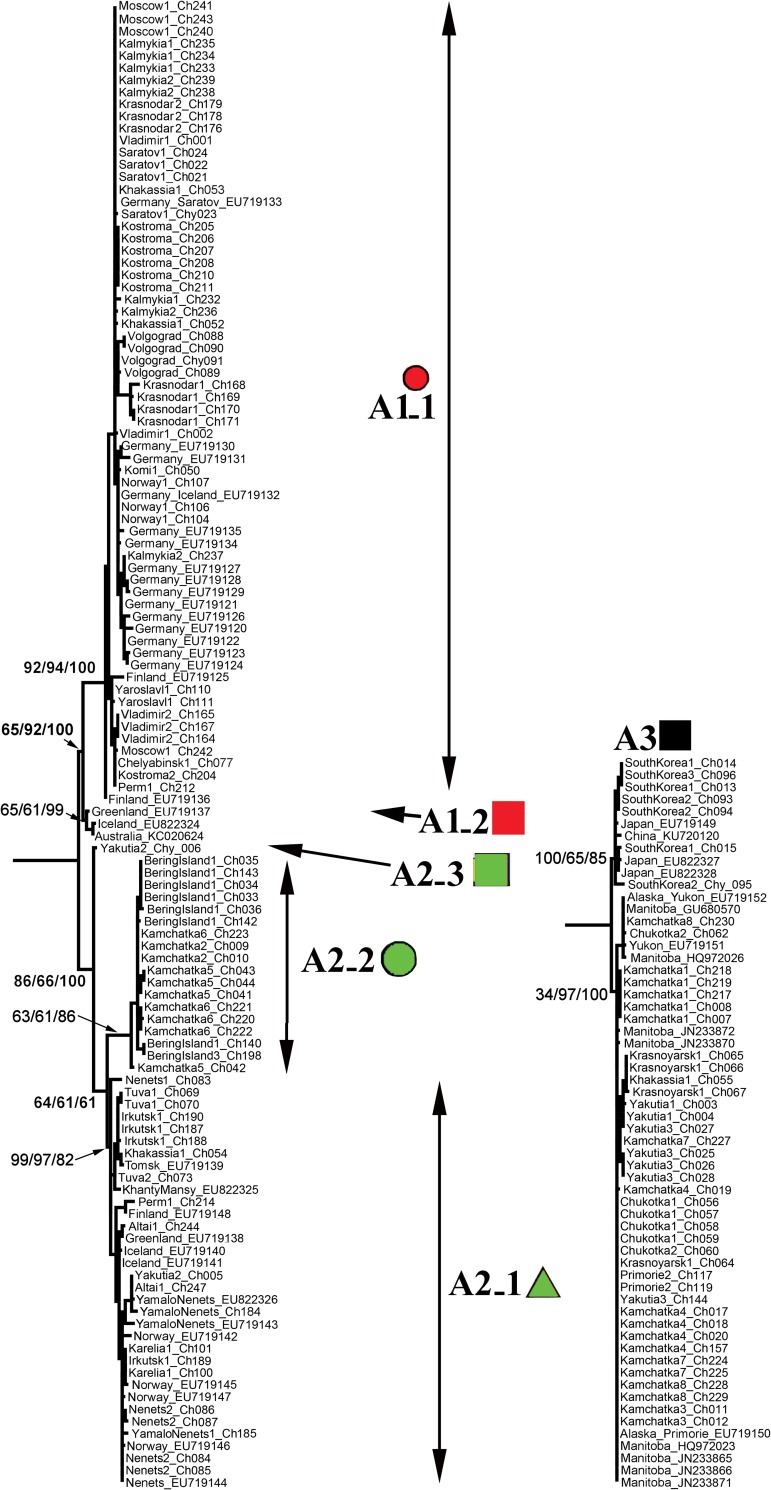
Complete subtree (collapsed in Fig 2) for the clades A1, A2 and A3 of the *Chydorus sphaericus* group showing support of the terminal clades. Geometric symbols for Eurasian taxa correspond to those in Fig 1.

The clade A1 (*Chydorus sphaericus* s.str.) is distributed in Europe and Western Siberia, with two sub-clades: widely distributed A1_1 and locally distributed A1_2 which has been recorded to date in Iceland and Greenland only (although it could be more widespread than is presently accepted)–A1_2 was introduced recently to Australia [77]. The clade A2 is present in Northern Europe and Western Siberia, and widely distributed in Eastern Siberia and Far East, including Kamchatka Peninsula and Bering Island. There are three sub-clades (Fig 3): widely distributed A2_1, A2_2 from Kamchatka and Bering Island only, and A2_3 represented to date by only a single population in Yakutia (Eastern Siberia).

The clade A3 is distributed in Far East of Russia, Eastern Siberia, Korea and Japan–genetic distances between specimens from very distant territories within this clade are very small.

### *ITS-2* gene phylogenetic tree

The best-fit model selected by jModeltest for the *ITS-2* fragment data set was Kimura 2-parameter (K80+G) with a gamma distribution with a lightest AIC weight. GARD detected no significant recombination breakpoints by either criterion used. The *ITS-2* sequence divergences (K80) varied between 0% and 23% among ingroup taxa.

**Fig 4:**
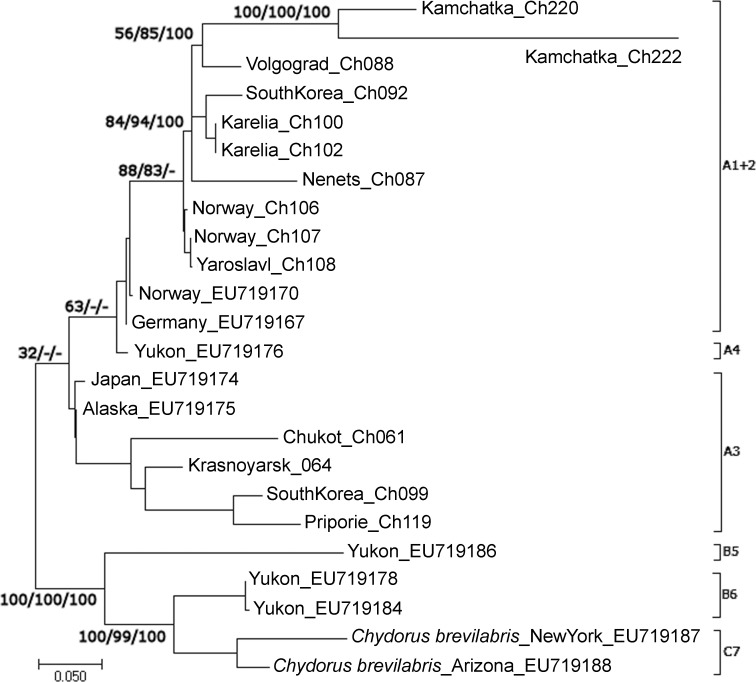
Maximum likehood tree based on sequences of nuclear rDNA internal transcribed spacer (*ITS-2*) gene representing the diversity among clades of the group A1+A2+A3 from the *Chydorus sphaericus* group. The support values of individual nodes are based on different variants of phylogenetic analysis: ML / ME / BI.

Only the group A1+A2+A3 is discussed here, because it contains our original sequences, see the discussion of other clades in Belyaeva & Taylor [7]. There are two main clades in this group: A3, and A1+A2 grouped in this tree with American clade A4 (Fig 4). But the support of such grouping is low, the nuclear gene does not support differentiation of the latter two mitochondrial clades. In this large group, the mitochondrial clade A2_3 is not represented (it was among bad sequences, see above), while the clade A2_2 forms a unique branch. Overall, the nuclear phylogeny provided even less resolution than the mitochondrial phylogeny.

### Demographic history: mitochondrial median haplotype network

**Fig 5:**
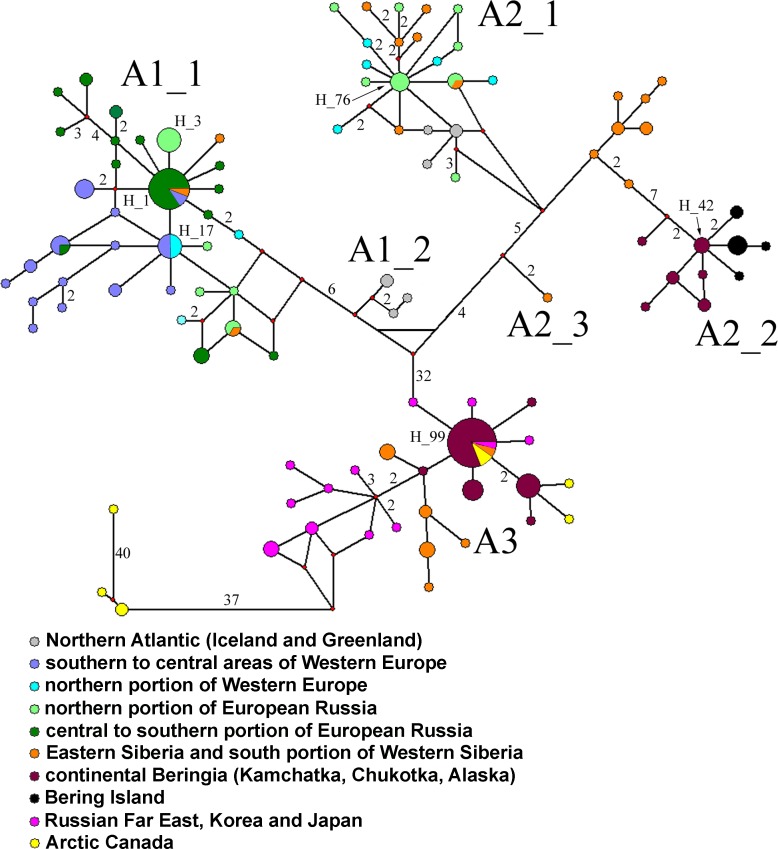
Median-joining cytochrome c oxidase subunit I (*COI*) haplotype network. Median vectors are indicated by small red circles. High-frequency haplotypes are labeled as well as the number of mutations for each branch (if not 1).

The median-joining network for 103 *COI* haplotypes (Fig 5) corroborated the ML and BI phylogenetic trees, but the A1 cluster is not monophyletic: clades A1_1 and A1_2 form unique groups. Although all A2 sub-clades form a monophyletic group, this clade is also clearly subdivided into three separate sub-clades, A2_1, A2_2 and A2_3, what also is an evidence of a strong isolation of the sub-clades of A2 phylogroup. The networks for the A1_1, A2_1, A2_2 and A3 clades showed star-like topology, suggesting recent population expansion events.

The center of the haplotype network for A1_1 clade is occupied by the haplotype H1, present in Germany, several regions of southern half to center of European Russia (Moscow Area, Vladimir Area, Saratov Area, Krasnodar Territory, Kalmykia Autonomous Republic) and Khakassia Autonomous Republic in SE portion of Western Siberia. A Haplotype H17 (from Germany and Norway) is in the center of a smaller star near H1.

The center of the haplotype network for A2_1 clade is occupied by the haplotype H76, present in the subarctic portion of European Russia (Nenets Autonomous Area and the Arkhangelsk Area). The center of the haplotype network for A2_2 clade is occupied by the haplotype H42, present in Kamchatka Peninsula only, but absent in Bering Island.

The center of the haplotype network for A3 clade is occupied by the haplotype H99, present in the Asian Beringia (Chukotka Peninsula, Kamchatka Peninsula), South of Russian Far East (Primorie Territory), Eastern Siberia (Krasnoyarsk Territory, Yakutia Autonomous Republic), American Beringia (Alaska) and Arctic Canada (Manitoba).

Tajima's D and mismatch distribution analysis supported the population expansion hypothesis for all these clades.

## Discussion

### Diversity and phylogeny of the *Chydorus sphaericus* group in the Northern Palearctic

Our study markedly expands and clarifies the biogeographic boundaries of the three large phylogroups [7] of the *Chydorus sphaericus* complex in Northern Eurasia. Continents are imperfect predictors of boundaries. Instead, several geographic boundaries are apparent within continents. Clades A1 and A3 (which also occurs in the western Nearctic), for example are broadly distributed in the Palearctic with a narrow geographic overlap at the center of the continent. While no new large clades were detected, regional subclades were found within the A1 and A2 clades. It is clear now that even a more narrow regionalism has persisted in this cladoceran species complex subsequent to the last glaciation as it was suggested before [7].

The existence of unique clades in the Eastern Palearctic has been detected in several other cladoceran groups [9, 78–80]. So the apparent increased diversity of this region seems relevant in both littoral and planktonic cladocerans. Interestingly, the transitional zone between the western A1 and eastern A3 clades occurs at the Yenisey River basin (grey shading in Fig 1). A similar genetic transition zone has been detected for the cladoceran genus *Moina* [10]. This region of the Palearctic is a candidate for a suture zone [81–82] for the cladoceran taxa, and even for all freshwater fauna. Surprisingly, specimens from three species (A1, A2_1 and A3) have been detected here in a single oxbow pond of the River Abakan, part of the Yenisey River system. It appears that the Yenisey River, separating Western and Eastern Siberia, is an approximate border line between eastern and western cladoceran faunistic zones–morphological evidence had previously supported this boundary [83]; see also the "line Baikal-Taimyr" [84].

It remains to be seen how taxonomically widespread the Palearctic boundary found here is in other freshwater zooplankton. Earlier it was concluded that the cladoceran fauna in Western Siberia is very similar to that of Europe [83–84], and some genetic data confirmed this opinion [85]. At the same time, some specific taxa were detected by morphological methods in the Eastern Siberia and preliminarily identified as "American" ones [50, 86]. The Eastern Siberian-Nearctic connection has been detected in previous genetic studies in other cladocerans [7, 78–80], but Eastern Siberia is still poorly represented. Thus far, only *Daphnia* of this region is just beginning to be studied with the use of molecular markers [87–88].

Our current findings mean that more work must be carried out to reconcile morphological and genetic information for the *Chydorus sphaericus* group. For example, Klimovsky & Kotov [50] conducted a morphological analysis of Central Yakutian populations and failed to detect *C*. cf. *sphaericus* while detecting two separate taxa: *C*. *belyaevae* Klimovsky & Kotov, 2015 and the *C*. cf. *biovatus*, previously found only in the Nearctic zone. Those findings need to be reconciled with the three clades that we detected here by a joint morphological-genetic study. Also we know little of the reproductive boundaries of the subclades that we discovered. These subclades can co-occur (e.g., A2_1 and A2_3 both occur in Yakutia) and so are not geographic subspecies. More nuclear markers and morphological study are required to test for reproductive boundaries in the overlap zone that we have identified. We have but a few sequences of the nuclear *ITS* gene, and we cannot exclude the possibility of hybridization between different phylogroups, as revealed in other groups of the Cladocera [89–93]. More efforts are necessary to reveal hybrids between different phylogroups of the *C*. *sphaericus* group, or to confirm their absence. No conflict of the mitochondrial and nuclear gene was detected by us, but (1) we have a limited number of sequences of the nuclear gene; (2) and in general *ITS*-2 lacks differentiation of the A2 and A1 clades.

### Phylogeography of the *Chydorus sphaericus* group in Northern Palearctic

Understanding the timescale of biogeographic events in the Cladocera has been severely limited by a dearth of fossils for calibrating molecular clocks (but see Kotov [15, 94]). Because the characters that separate the clades (male morphology and number of resting eggs) are unlikely to be detected in the fossil or in the Holocene subfossil record [95–96], calibration options for the *Chydorus sphaericus-brevilabris* complex remain poor. Calibrations from mutation accumulation experiments are rare and those for the Cladocera appear to result in severe underestimates for deeper divergences [97]. Belyaeva & Taylor [7] calibrated a molecular clock for the clades of *Chydorus* studied here based on a general arthropod calibration for 16S rRNA molecular clocks. They estimated the common ancestor of the clade A1+A2 as 2–10 MYA, from the late Pliocene–early Pleistocene, and the common ancestor of clades A1+A2 and A3 to be 5–15 MYA, from the late Miocene. Dates estimated with calibrations from other invertebrate lineages can also have pronounced error, i.e. a ten-fold difference between sister clades can occur [98]. Also, such rates decrease as a function of time [99–101].

Even with the error of the existing molecular clock estimations we can be reasonably certain that clades A2_1, A2_2 and A2_3, are younger than the geological events that disrupted the supercontinents [11, 102] and caused the mass extinction of the cladoceran fauna in the middle Caenozoic [103]. But, it is clear from our study that the groups were old enough to have been affected by Pleistocene climatic oscillations.

The star-like patterns found in the mitochondrial haplotype network may be due to recent postglacial recolonization events [92, 98, 104]. Such star-like patterns in *C*. *sphaericus* group are obviously associated with very distant regions of Eurasia. The network suggests that the complex survived recent glaciation in separate refugia. H1, the central haplotype of the A1_1, clade is distributed predominantly in the territories of Europe, affected by glaciation during the Quaternary maximum, but not glaciated during the Last Glacial Maximum [105]. Presumably, the A1 phylogroup survived in a refugium in the southern portion of European Russia (and, probably, in the southern portion of "non-Russian" Europe, which is poorly sampled).

Interpretation of the fate of A2 phylogroup is more complicated. The central haplotype of A2_1 is present now in the territory (Nenets Autonomous Area and Arkhangelsk Area) with a complicated geological history. This region was covered by an ice shield in the Middle Pleistocene [105–106]. According to the most modern reconstructions [106], the region was covered by an ice sheet in the Early Weichselian, but free of ice in the Late Weichselian. Earlier authors proposed contrasting reconstructions [105–108]. But, in any case some "cryptic northern refugia" [109–110] may have existed for A2_1 in the late Pleistocene. Remarkably, distinct mitochondrial lineages of the *Daphnia longispina* complex were found in the same area, which also suggests the existence of a local/regional refugium in this area [111].

The Weichselian ice sheet was associated with huge proglacial lakes (ice-dammed lakes) whose drainage changed from south-north to a north-south direction [112–113]. Such water movement may have enabled dispersal of cladocerans in a southern direction. Our data demonstrate that this region of proglacial lakes may have acted a source for recolonization. A similar scenario has been proposed for the cladoceran genus *Polyphemus* by Xu et al. [79].

The interpretation of biogeography of clades A1_2 (Greenland and Iceland) and A2_3 (Eastern Siberia), as well as some other small clades (i.e. East Siberian–with upper position in our network–branch of the subclade of A2_2), is not so obvious. They could be relict clades, largely erased by most recent Pleistocene glacial cycle(s), but testing of such hypothesis requires further studies, i.e. intensive sampling in Greenland, Iceland and Eastern Siberia.

The A2_2 and A3 phylogroups appear to have survived in the Beringian refugium. Therefore, a northern refugium was very important for survival of diversity in the complex. The Beringian refugium has been proposed for many other species of cladocerans [79, 114] and other freshwater and terrestrial animals [82, 115–116].

It is remarkable that the inferred central haplotype of the A2_2 clade remains undetected on Bering Island, which has a very scarce cladoceran fauna [117]. According to our network, this island was colonized independently by several (at least three) lineages. Although Bering Island is located relatively close to Kamchatka Peninsula, it is separated by a very deep strait. Bering Island has existed at least since the Eocene [118], and was never incorporated into the Beringian land bridge. Therefore, the dispersal hypothesis appears to be supported by the geological history of this region. Earlier it was shown that this island was also colonized by the cladocerans of the genus *Eurycercus* from the Nearctic, possibly by migratory waterfowl [119]. *Chydorus* cf. *sphaericus* of the clade A3 may have colonized Bering Island by the same mode.

## Conclusions

We found six different phylogroups (A1_1, A1_2, A2_1, A2_2, A2_3 and A3) of the *C*. *sphaericus* group with different phylogeographic histories in northern Eurasia. Among them four (A1_1, A2_1, A2_2 and A3) passed through a relatively recent population expansion, probably associated with the recolonization of territories previously affected by glaciation. Europe was an apparent source for recolonization of two clades (A1 and A2_1), and Beringia for two other clades (A2_2 and A3). The central portion of Eurasia (the Yenisey River basin) appears to be a zone of secondary contact for European and Beringian phylogroups, where we detected their co-occurrence in the same water body. This zone may prove useful in testing reproductive boundaries between clades that had been previously isolated in different refugia. Our results reveal that the northern Palearctic is an important region for understanding the evolutionary outcomes and recolonization routes following Pleistocene glaciation for Holarctic freshwater invertebrates.

## Supporting Information

S1 Table Complete list of sequences obtained in this study with information on locality and the GenBank accession for *COI* and *ITS-2* sequences number provided for each specimen. Clade designations correspond to those in other tables. AR in list of states = Autonomous Republic.
(DOC)

S2 Table List of sequences from the GenBank that were used in our study. (DOC)

## References

[1] Forró L, Korovchinsky NM, Kotov AA, Petrusek A. Global diversity of cladocerans (Cladocera; Crustacea) in freshwater. Hydrobiologia. 2008;595: 177–184.

[2] Adamowicz SJ, Petrusek A, Colbourne JK, Hebert PDN, Witt JDS. The scale of divergence: a phylogenetic appraisal of intercontinental allopatric speciation in a passively dispersed freshwater zooplankton genus. Mol Phylogenet Evol. 2009;50: 423–436. 10.1016/j.ympev.2008.11.026 19124080

[3] Galimov Y, Walser B, Haag CR. Frequency and inheritance of non-male producing clones in *Daphnia magna*: evolution towards sex specialization in a cyclical parthenogen? J Evol Biol. 2011;24: 1572–1583. 10.1111/j.1420-9101.2011.02288.x 21599772

[4] Crease TJ, Omilian AR, Costanzo KS, Taylor DJ. Transcontinental phylogeography of the *Daphnia pulex* species complex. PLoS ONE. 2012;7(10): e46620 10.1371/journal.pone.0046620 23056371 PMC3463573

[5] Petrusek A, Černý M, Audenaert E. Large intercontinental differentiation of *Moina micrura* (Crustacea: Anomopoda): one less cosmopolitan cladoceran? Hydrobiologia. 2004;526: 73–81.

[6] Sacherová V., Hebert PDN. The evolutionary history of the Chydoridae (Crustacea: Cladocera). Biol J Linn Soc. 2003;79: 629–643.

[7] Belyaeva M, Taylor DJ. Cryptic species within the *Chydorus sphaericus* species complex (Crustacea: Cladocera) revealed by molecular markers and sexual stage morphology. Mol Phylogenet Evol. 2009;50: 534–546. 10.1016/j.ympev.2008.11.007 19049884

[8] Kotov AA, Ishida S, Taylor DJ. Revision of the genus *Bosmina* Baird, 1845 (Cladocera: Bosminidae), based on evidence from male morphological characters and molecular phylogenies. Zool J Linn Soc. 2009;156: 1–56.

[9] Bekker EI, Kotov AA, Taylor DJ. A revision of the subgenus *Eurycercus* (*Eurycercus*) Baird, 1843 emend. nov. (Cladocera: Eurycercidae) in the Holarctic with the description of a new species from Alaska. Zootaxa. 2012;3206: 1–40.

[10] Bekker EI, Karabanov DP, Galimov YR, Kotov AA. DNA barcoding reveals high cryptic diversity in the North Eurasian *Moina* species (Crustacea: Cladocera). PloS ONE. 2016;11: e0161737 10.1371/journal.pone.0161737 27556403 PMC4996527

[11] Fryer G. Phylogeny and adaptive radiation within the Anomopoda: a preliminary exploration. Hydrobiologia. 1995;307: 57–68.

[12] Incagnone G, Marrone F, Barone R, Robba L, Naselli-Flores L. How do freshwater organisms cross the “dry ocean”? A review on passive dispersal and colonization processes with a special focus on temporary ponds. Hydrobiologia. 750;2014: 103–123.

[13] Ortells R, J. Vanoverbeke GL, De Meester L. Colonization of *Daphnia magna* in a newly created pond: founder effects and secondary immigrants. Hydrobiologia. 2014;723: 167–179.

[14] Fryer G. Observations on the ephippia of certain macrothricid cladocerans. Zool J Linn Soc. 1972;51: 79–96.

[15] Kotov AA. Morphology and phylogeny of the Anomopoda (Crustacea: Cladocera). Moscow: KMK Press; 2013.

[16] Müller OF. Zoologiae Danicae Prodromus, seu Animalium Daniae et Norvegiae Indigenarum characteres, nomina et synonyma imprimis popularium Havniae: typis Hallageriis; 1776.

[17] Leach WE. Annulosa. Classe I.–Crustacea Milar J, editor. Supplement to the 4th edition of Encyclopedia Britannica. Edinburgh: Archibald Constable; 1816 p. 401.

[18] King RL. On Australian Entomostraca–in continuation. Pap Proc Roy Soc Tasm. 1853;2: 253–263.

[19] Schödler JE. Die Lynceiden und Polyphemiden der Umgebung von Berlin. Jahr Dorotheen Realschule Berlin, 1862: 1–126.

[20] Poggenpol M. Spisok Copepoda, Cladocera i Ostracoda Mockvy i blizhaishikh ee okrestnostei. Izv Imper Obshch Lyubit Estestvozn Antropolog Etnogr. 1874;10: 69–77.

[21] Sars GO. Oversigt af Norges Crustaceer, med foreløbige bemærkninger over de nye eller mindre bekjendte Arter. II. (Branchiopoda—Ostracoda—Cirripedia). Forhand Videns–Selsk Christian. 1890;1: 1–80.

[22] Birge EA. Notes on Cladocera. III. Trans Wisconsin Acad Sci, Arts & Letters. 1893;9: 275–317.

[23] Daday E. Mikroskopische Süsswasserthiere aus Ceylon. Termész Füzetek. 1898;21: 1–123.

[24] Sars GO. Contributions to the knowledge of the fresh-water Entomostraca of South America, as shown by artificial hatching from dried material. 1. Cladocera. Arch Math Naturvidensk. 1901;23: 1–102.

[25] Daday E. Untersuchungen über die Süsswasser Mikrofauna Paraguays. Zoologica, Ser 18. 1905;44: 1–374.

[26] Stingelin T. Untersuchungen über die Cladocerenfauna von Hinterindien, Sumatra und Java, nebst einem Beitrag zur Cladoceren-Kenntnis der Hawaii-Inseln. Zool Jahrb Abt Syst. 1905;21: 327–367.

[27] Henry M. On some Australian Cladocera. J Proc Roy Soc NSW. 1919;52: 463–485.

[28] Flössner D. Krebstiere, Crustacea (Kiemen- und Blattfüßer, Branchiopoda, Fischläuse, Branchiura). Die Tierwelt Deutschlands. 1972;60: 1–499.

[29] Flössner D. Die Haplopoda und Cladocera (ohne Bosminidae) Mitteleuropas. Leiden: Backhuys; 2000.

[30] Smirnov NN. Chydoridae fauni mira. Fauna SSSR. Rakoobraznie. 1971;1(2): 1–531.

[31] Frey DG. On the plurality of *Chydorus sphaericus* (O. F. Müller) (Cladocera, Chydoridae) and designation of a neotype from Sjaelsø, Denmark. Hydrobiologia. 1980;69: 83–123.

[32] Frey DG. Contrasting strategies of gamogenesis in northern and southern populations of Cladocera. Ecology. 1982;63: 223–241.

[33] Frey DG. Questions concerning cosmopolitanism in Cladocera. Arch Hydrobiol. 1982;93: 484–502.

[34] Frey DG. Relocation of *Chydorus barroisi* and related species (Cladocera, Chydoridae) to a new genus and description of two new species. Hydrobiologia. 1982;86: 231–269.

[35] Frey DG. The honeycombed species of *Chydorus* (Cladocera, Chydoridae): comparison of *C*. *bicornutus* and C. *bicollaris* n. sp. with some preliminary comments on *faviformis*. Can J Zool. 1982;60: 1892–1916.

[36] Frey DG. The reticulated species of *Chydorus* (Cladocera, Chydoridae): two new species with suggestions of convergence. Hydrobiologia. 1982;93: 255–279.

[37] Frey DG. A new species of the *Chydorus sphaericus* group (Cladocera, Chydoridae) from Western Montana. Int Rev ges Hydrobiol. 1985;70: 3–20.

[38] Frey DG. The North American *Chydorus faviformis* (Cladocera, Chydoridae) and the honeycombed taxa of other continents. Philos Trans R Soc Lond B Biol Sci. 1987;315: 353–402.

[39] Frey DG. The non-cosmopolitanism of chydorid Cladocera: implications for biogeography and evolution In: Gore RH, Heck KL, editors. Crustacean Biogeography (Crustacean issues 4). Rotterdam: A.A. Balkema; 1987 pp. 237–256.

[40] Frey DG. The taxonomy and biogeography of the Cladocera. Hydrobiologia. 1987;145: 5–17.

[41] Paggi JC. Nota sistemática acerca de algunos cladóceros del género *Chydorus* Leach 1843, de la República Argentina. Physis. 1972;31: 223–236.

[42] Chengalath R, Hann BJ. A new species of *Chydorus* (Cladocera: Chydoridae) from Ontario, Canada. Trans Am Microsc Soc. 1981;100: 229–238.

[43] Chen SZ. Redescription of *Chydorus undulatus* Chiang (Crustacea, Cladocera) and note on its ecology. Acta Hydrobiol Sin. 1984;8: 341–344.

[44] Rajapaksa R, Fernando CH. A review of the systematics and distribution of *Chydorus ventricosus* Daday, 1898, with the first description of the male and redescription of the species. Can J Zool. 1986;64: 818–832.

[45] Røen U. *Chydorus arcticus* n.sp., a new cladoceran crustacean (Chydoridae: Chydorinae) from the North Atlantic Arctic and Subarctic areas. Hydrobiologia. 1987;145: 125–130.

[46] Alonso M. *Chydorus pizarri* sp. nov. a new chydorid (Cladocera) from Western Spain. Limnetica. 1988;4: 27–40.

[47] Alonso M. Crustacea, Branchiopoda. Fauna Iberica 7 Crustacea Branchiopoda. Madrid: Museo Nacional de Ciencias Naturales, Consejo Superior de Investigaciones Científicas; 1996.

[48] Smirnov NN, Sheveleva NG. *Chydorus irinae* sp. n. (Anomopoda, Chydoridae, Chydorinae) from the Tom’ River (the Amur basin, Russia). Zool Zh. 2010; 89: 635–638.

[49] Van Damme K, Dumont HJ. Cladocera of the Lençóis Maranhenses (NE–Brazil): faunal composition and a reappraisal of Sars’ method. Braz J Biol. 2010;70: 755–779. 21085782 10.1590/s1519-69842010000400008

[50] Klimovsky AI, Kotov AA. Cladocera (Crustacea, Branchiopoda) of Central Yakutia 3. Taxa from the *Chydorus sphaericus* s. l. species group (Anomopoda, Chydoridae). Zool Zh. 2015;94: 1257–1267.

[51] Hudec I. Anomopoda, Ctenopoda, Haplopoda, Onychopoda (Crustacea: Branchiopoda) Fauna Slovenska III. Bratislava: VEDA; 2010.

[52] Alonso M. *Estatheroporus gauthieri*, new genus, new species (Cladocera: Chydoridae), from Mediterranean countries. J Crustacean Biol. 1990;10: 148–161.

[53] DeWaard JR, Sacherova V, Cristescu MEA, Remigio EA, Crease TJ, Hebert PDN. Probing the relationships of the branchiopod crustaceans. Mol Phylogenet Evol. 2006;39: 491–502. 10.1016/j.ympev.2005.11.003 16406819

[54] Costa FO, DeWaard JR, Boutillier J, Ratnasingham S, Dooh RT, Hajibabaei M, et al Biological identifications through DNA barcodes: the case of the Crustacea. Can J Fish Aquat Sci. 2007;64: 272–295.

[55] Elías-Gutiérrez M, Martínez Jerónimo F, Ivanova NV, Valdez Moreno M, Hebert PDN. DNA barcodes for Cladocera and Copepoda from Mexico and Guatemala, highlights and new discoveries. Zootaxa. 2008;1839: 1–42.

[56] Jeffery NW, Elías-Gutiérrez M, Adamowicz SJ. Species diversity and phylogeographical affinities of the Branchiopoda (Crustacea) of Churchill, Manitoba, Canada. PLoS ONE. 2011;6(5): e18364 10.1371/journal.pone.0018364 21610864 PMC3096620

[57] Prosser S, Martínez-Arce A, Elías-Gutiérrez M. A new set of primers for *COI* amplification from freshwater microcrustaceans. Mol Ecol Res. 2013;13: 1151–1155. 10.1111/1755-0998.12132 23795700

[58] Taylor DJ, Ishikane CR, Haney RA. The systematics of Holarctic bosminids and a revision that reconciles molecular and morphological evolution. Limnol Oceanogr. 2002;47: 1486–1495.

[59] Omilian AR, Taylor DJ. Rate acceleration and long-branch attraction in a conserved gene of cryptic Daphniid (Crustacea) species. Mol Biol Evol. 2001;18: 2201–2212. 11719570 10.1093/oxfordjournals.molbev.a003767

[60] Okonechnikov K, Golosova O, Fursov M. Unipro UGENE: a unified bioinformatics toolkit. Bioinformatics. 2012;28: 1166–1167. 10.1093/bioinformatics/bts091 22368248

[61] Sievers F, Wilm A, Dineen D, Gibson TJ, Karplus K, Li W, et al Fast, scalable generation of high-quality protein multiple sequence alignments using Clustal Omega. Mol Syst Biol. 2011;7: 539 10.1038/msb.2011.75 21988835 PMC3261699

[62] Librado P, Rozas J. DnaSP v5: a software for comprehensive analysis of DNA polymorphism data. Bioinformatics. 2009;25: 1451–1452. 10.1093/bioinformatics/btp187 19346325

[63] Kumar S, Stecher G, Tamura K. MEGA7: Molecular Evolutionary Genetics Analysis version 7.0 for bigger datasets. Mol Biol Evol. 2016;33: 1870–1874. 10.1093/molbev/msw054 27004904 PMC8210823

[64] Tajima F. Statistical method for testing the neutral mutation hypothesis by DNA polymorphism. Genetics. 1989;123: 585–595. 2513255 10.1093/genetics/123.3.585 PMC1203831

[65] Fu Y. Statistical tests of neutrality of mutations against population growth, hitchhiking and background selection. Genetics. 1997;147: 915–925. 9335623 10.1093/genetics/147.2.915 PMC1208208

[66] Excoffier L, Lischer HEL. Arlequin suite ver 3.5: a new series of programs to perform population genetics analyses under Linux and Windows. Mol Ecol Res. 2010;10: 564–567. 10.1111/j.1755-0998.2010.02847.x 21565059

[67] Rogers AR, Harpending H. Population growth makes waves in the distribution of pairwise genetic differences. Mol Biol Evol. 1992;9: 552–569. 1316531 10.1093/oxfordjournals.molbev.a040727

[68] Darriba D, Taboada G, Doallo R, Posada D. jModelTest 2: more models, new heuristics and parallel computing. Nature Meth. 2012;9: 772. 10.1038/nmeth.2109 PMC4594756 22847109

[69] Posada D, Buckley T. Model selection and model averaging in phylogenetics: advantages of Akaike Information Criterion and Bayesian approaches over likelihood ratio tests. Syst Biol. 2004;53: 793–808. 10.1080/10635150490522304 15545256

[70] Tamura K. Estimation of the number of nucleotide substitutions when there are strong transition-transversion and G+C-content biases. Mol Biol Evol. 1992;9: 678–687. 1630306 10.1093/oxfordjournals.molbev.a040752

[71] Kimura MA. Simple method for estimating evolutionary rate of base substitutions through comparative studies of nucleotide sequences. J Mol Evol. 1980;16: 111–120. 7463489 10.1007/BF01731581

[72] Swofford D. PAUP*. Phylogenetic Analysis Using Parsimony (* and other methods). Version 4. Sunderland (MA): Sinauer Associates; 2003.

[73] Ronquist F, Teslenko M, van der Mark P, Ayres DL, Darling A, Höhna S, et al MrBayes 3.2: Efficient Bayesian phylogenetic inference and model choice across a large model space. Syst Biol. 2012;6: 539–542. 10.1093/sysbio/sys029 PMC3329765 22357727

[74] Bandelt H-J, Forster P, Röhl A. Median-joining networks for inferring intraspecific phylogenies. Mol Biol Evol. 1999;16: 37–48. 10331250 10.1093/oxfordjournals.molbev.a026036

[75] Kosakovsky Pond SL, Posada D, Gravenor MB, Woelk CH, Frost SD. Automated phylogenetic detection of recombination using a genetic algorithm. Mol Biol Evol. 2006;23: 1891–1901. 10.1093/molbev/msl051 16818476

[76] Delport W, Poon AF, Frost SD, Kosakovsky Pond SL. Datamonkey 2010: a suite of phylogenetic analysis tools for evolutionary biology. Bioinformatics. 2010;26: 2455–2457. 10.1093/bioinformatics/btq429 20671151 PMC2944195

[77] Sharma P, Kotov AA. Establishing of *Chydorus sphaericus* (O.F. Muller) s.str. (Crustacea: Cladocera) in Australia: consequences of mass fish stock from Northern Europe? J Limnol. 2015;74: 225–233.

[78] Ishida S, Taylor DJ. Mature habitats associated with genetic divergence despite strong dispersal ability in an arthropod. BMC Evol Biol. 2007;7: 52 10.1186/1471-2148-7-52 17407568 PMC1852300

[79] Xu S, Hebert PDN, Kotov AA, Cristescu ME. The non-cosmopolitanism paradigm of freshwater zooplankton: insights from the global phylogeography of the predatory cladoceran *Polyphemus pediculus* (Crustacea, Onychopoda). Mol Ecol. 2009;18: 5161–5179. 10.1111/j.1365-294X.2009.04422.x 19912535

[80] Millette KL, Xu S, Witt JDS, Cristescu ME. Pleistocene-driven diversification in freshwater zooplankton: Genetic patterns of refugial isolation and postglacial recolonization in *Leptodora kindtii* (Crustacea, Cladocera). Limnol Oceanogr. 2011;56: 1725–1736.

[81] Remington CL. Suture-zones of hybrid interaction between recently joined biotas. Evol Biol. 1968;2: 321–428.

[82] Hewitt GM. The structure of biodiversity–insights from molecular phylogeography. Front Zool. 2004;1: 4 10.1186/1742-9994-1-4 15679920 PMC544936

[83] Kotov AA. Faunistic complexes of the Cladocera (Crustacea, Branchiopoda) of Eastern Siberia and Far East of Russia. Zool Zh. 2016;95: 748–768.

[84] Korovchinsky NM. Cladocerans of the order Ctenopoda of the world fauna (morphology, systematics, ecology, biogeography) Moscow: KMK Press; 2004.

[85] Ishida S, Taylor D.J. Quaternary diversification in a sexual Holarctic zooplankter, *Daphnia galeata*. Mol Ecol. 2007b;16: 569–582. 17257114 10.1111/j.1365-294X.2006.03160.x

[86] Klimovsky AI, Bekker EI, Sinev AY, Korovchinsky NM, Smirnov NN, Kotov АА. Cladocera (Crustacea, Branchiopoda) of Central Yakutia. 4. Taxonomical-faunistic and zoogeographical analysis. Zool Zh. 2015;94: 1367–1378.

[87] Zuykova EI, Bochkarev NA, Katokhin AV. Identification of the *Daphnia species* (Crustacea: Cladocera) in the lakes of the Ob and Yenisei River basins: morphological and molecular phylogenetic approaches. Hydrobiologia. 2012;715: 135–150.

[88] Zuykova EI, Bochkarev NA, Sheveleva NG. Genetic polymorphism, haplotype distribution, and phylogeny of *Daphnia* (Cladocera: Anomopoda) species from the water bodies of Russia as inferred from the 16S mtDNA gene sequencing. Russ J Genet. 2016;52: 585–596. 29368496

[89] Schwenk K. Interspecific hybridization in *Daphnia*: distinction and origin of hybrid matrilines. Mol Biol Evol. 1993;10: 1289–1302. 8277855 10.1093/oxfordjournals.molbev.a040076

[90] Dlouhá S, Thielsch A, Kraus RHS, Seda J, Schwenk K, Petrusek A. Identifying hybridizing taxa within the *Daphnia longispina* species complex: a comparison of genetic methods and phenotypic approaches. Hydrobiologia. 2010;643: 107–122.

[91] Ishida S, Takahashi A, Matsushima N, Yokoyama J, Makino W, Urabe J, et al The long-term consequences of hybridization between the two *Daphnia* species, *D*. *galeata* and *D*. *dentifera*, in mature habitats. BMC Evol Biol. 2011; 11: 209 10.1186/1471-2148-11-209 21756366 PMC3156774

[92] Xu S, Innes DJ, Lynch M, Cristescu M.E. The role of hybridization in the origin and spread of asexuality in *Daphnia*. Mol Ecol. 2013;22: 4549–4561 10.1111/mec.12407 23879327 PMC3902101

[93] Litvinchuk LF, Litvinchuk SN. Morphological diversity and widespread hybridization in the genus *Bythotrephes* Leydig, 1860 (Branchiopoda, Onychopoda, Cercopagidae). Arch Biol Sci. 2016;68: 1–25.

[94] Kotov AA. Jurassic Cladocera (Crustacea, Branchiopoda) with a description of an extinct Mesozoic order. J Nat Hist. 2007;41: 13–37.

[95] Szeroczyńska K, Sarmaja-Korjonen K. Atlas of subfossil Cladocera from Central and Northern Europe. Świecie: Friends of the Lower Vistula Society; 2007.

[96] Korosi JB, Smol JP. An illustrated guide to the identification of cladoceran subfossils from lake sediments in northeastern North America: part 2 –the Chydoridae. J. Paleolimnol. 2012;48: 587–622.

[97] Kotov AA, Taylor DJ. Mesozoic fossils (>145 Mya) suggest the antiquity of the subgenera of *Daphnia* and their coevolution with chaoborid predators. BMC Evol Biol. 2011;11:129 10.1186/1471-2148-11-129 21595889 PMC3123605

[98] Bolotov IN, Aksenova JV, Bespalaya YV, Gofarov MY, Kondakov AV, Paltser IS, et al Origin of a divergent mtDNA lineage of a freshwater snail species, *Radix balthica*, in Iceland: cryptic glacial refugia or a postglacial founder event? Hydrobiologia. 2016;

[99] Ho SY, Phillips MJ, Cooper A, Drummond AJ. Time dependency of molecular rate estimates and systematic overestimation of recent divergence times. Mol Biol Evol. 2005;22: 1561–1568. 10.1093/molbev/msi145 15814826

[100] Ho SY, Tong KJ, Foster CS, Ritchie AM, Lo N, Crisp MD. Biogeographic calibrations for the molecular clock. Biol Lett. 2015;11: 20150194 10.1098/rsbl.2015.0194 26333662 PMC4614420

[101] Molak M, Ho SY. Prolonged decay of molecular rate estimates for metazoan mitochondrial DNA. PeerJ. 2015;3: e821 10.7717/peerj.821 25780773 PMC4358697

[102] Benzie JAH. The biogeography of Australian *Daphnia*: clues of an ancient (> 70 m.y.) origin for the genus. Hydrobiologia. 1987;145: 51–65.

[103] Korovchinsky NM. The Cladocera (Crustacea: Branchiopoda) as a relict group. Zool J Linn Soc. 2006;147: 109–124.

[104] Avise JC. Phylogeography: The History and Formation of Species. Cambridge: Harvard University Press, 2000.

[105] Svendsen J, Alexanderson H, Astakhov V, Demidov I, Dowdeswell J, Funder S., et al Late quaternary ice sheet history of Northern Eurasia. Quat Sci Rev. 2004;23: 1229–1271.

[106] Astakhov VI. Pleistocene glaciations of northern Russia–a modern view. Boreas. 2013;42: 1–24.

[107] Ehlers J, Gibbard P. Extent and chronology of Quaternary glaciation. Episodes. 2008;31: 211–218.

[108] Andreicheva LN, Marchenko-Vagapova TI. The Neopleistocene of North European Russia: Stratigraphy, paleogeography, and paleoclimate. Stratigr Geol Correl. 2007;15: 421–436.

[109] Stewart JR., Lister AM. Cryptic northern refugia and the origins of the modern biota. Trends Ecol Evol. 2001;16: 608–613.

[110] Stewart JR, Lister AM, Barnes I, Dalén L. Refugia revisited: individualistic responses of species in space and time. Proc R Soc B. 2010;277: 661–671. 10.1098/rspb.2009.1272 19864280 PMC2842738

[111] Petrusek A, Thielsch A, Schwenk K. Mitochondrial sequence variation suggests extensive cryptic diversity within the Western Palearctic *Daphnia longispina* complex. Limnol. Oceanogr. 2012;57: 1838–1845.

[112] Mangerud J, Astakhov V, Jakobsson M, Svendsen J. Huge Ice-age lakes in Russia. J Quat Sci. 2001;16:773–777.

[113] Astakhov VI. Evidence of Late Pleistocene ice-dammed lakes in West Siberia. Boreas. 2006; 35: 607–621.

[114] Hebert PDN, Rowe CL, Adamowicz SJ. Life at low temperatures: A novel breeding-system adjustment in a polar cladoceran. Limnol Oceanogr. 2007;52; 2507–2518.

[115] Hewitt GM. Genetic consequences of climatic oscillations in the Quaternary. Phil Trans R Soc B. 2004;359: 183–195. 10.1098/rstb.2003.1388 15101575 PMC1693318

[116] Wooller MJ, Gaglioti B, Fulton TL, Lopez A, Shapiro B. Post-glacial dispersal patterns of Northern pike inferred from an 8800 year old pike (*Esox* cf. *lucius*) skull from interior Alaska. Quat Sci Rev. 2015;120: 118–125.

[117] Novichkova AA, Chertoprud ES. The freshwater crustaceans (Cladocera: Copepoda) of Bering Island (Commander Islands, Russian Far East): species richness and taxocene structure. J Nat Hist. 2016;50: 1357–1368.

[118] Tsvetkov AA, Fedorchuk AV, Gladenkov AY. Geology and magmatic evolution of Bering island. Intern Geol Rev. 1990;32: 1202–1217.

[119] Bekker EI, Novichkova AA, Kotov AA. New findings of *Eurycercus* Bairs, 1843 (Cladocera: Anomopoda) in the Eastern Palaearctic. Zootaxa. 2014;3895: 297–300. 10.11646/zootaxa.3895.2.11 25543572

