# Supplementary material for: Phylogeography of the Chydorus sphaericus Group (Cladocera: Chydoridae) in the Northern Palearctic
Source: PLoS One. 2016 Dec 19;11(12):e0168711. doi: 10.1371/journal.pone.0168711 (PMC5167426; doi:10.1371/journal.pone.0168711)
Supplement: S1 Table — Clade designations correspond to those in other tables. AR in list of states = Autonomous Republic. (DOC) [file pone.0168711.s001.doc]

**S1 Table.** Complete list of sequences obtained in this study with information on locality and the GenBank accession for *COI* and *ITS-2* sequences number provided for each specimen. Clade designations correspond to those in other tables. AR in list of states = Autonomous Republic.

| **NCBI - COI** | **NCBI - ITS** | **Clade** | **Seq unique ID** | **Country** | **State** | **Locality** | **N** | **E** | **Sample number** |
| --- | --- | --- | --- | --- | --- | --- | --- | --- | --- |
| KX431587 |  | A1 | Ch1 | Russia (European) | Vladimir Area | Klyazma River | 55.8998 | 39.479 | AAK M-2734 |
| KX431645 |  | A1 | Ch2 | Russia (European) | Vladimir Area | Klyazma River | 55.8998 | 39.479 | AAK M-2734 |
| KX431605 |  | A3 | Ch3 | Russia (Asian) | Yakutia Autonomous Republic | A puddle on the North of Samoilovsky Island | 72.387 | 126.4826 | AAK M-1902 |
| KX431606 |  | A3 | Ch4 | Russia (Asian) | Yakutia Autonomous Republic | A puddle on the North of Samoilovsky Island | 72.387 | 126.4826 | AAK M-1902 |
| KX431623 |  | A2_1 | Ch5 | Russia (Asian) | Yakutia Autonomous Republic | A small lake at cemetery, near village of Miuri, region of Bogorontsi | 62.629 | 130.095 | AAK M-2323 |
| KX431624 |  | A2_3 | Ch6 | Russia (Asian) | Yakutia Autonomous Republic | A small lake at cemetery, near village of Miuri, region of Bogorontsi | 62.629 | 130.095 | AAK M-2323 |
| KX431657 |  | A3 | Ch7 | Russia (Asian) | Kamchatka Area | A small lake in the caldera of Uzon volcano, Kronotsky State Natural Reserve | 54.49925 | 159.9903 | AAK M-3003 |
| KX431657 |  | A3 | Ch8 | Russia (Asian) | Kamchatka Area | A small lake in the caldera of Uzon volcano, Kronotsky State Natural Reserve | 54.49925 | 159.9903 | AAK M-3003 |
| KX431661 |  | A2_2 | Ch9 | Russia (Asian) | Kamchatka Area | A partly dried affluent of the Shumnaya River, Kronotsky State Natural Reserve | 54.30375 | 160.2887 | AAK M-3036 |
| KX431661 |  | A2_2 | Ch10 | Russia (Asian) | Kamchatka Area | A partly dried affluent of the Shumnaya River, Kronotsky State Natural Reserve | 54.30375 | 160.2887 | AAK M-3036 |
| KX431603 |  | A3 | Ch11 | Russia (Asian) | Kamchatka Area | A small puddle, vicinities of Kronoskoe Lake, Kronotsky State Natural Reserve | 54.69062 | 160.3211 | AAK M-3052 |
| KX431603 |  | A3 | Ch12 | Russia (Asian) | Kamchatka Area | A small puddle, vicinities of Kronoskoe Lake, Kronotsky State Natural Reserve | 54.69062 | 160.3211 | AAK M-3052 |
| KX431627 |  | A3 | Ch13 | South Korea |  | Gae Biulgol Reservoir | 36.56528 | 127.0189 | AAK M-2382 |
| KX431627 |  | A3 | Ch14 | South Korea |  | Gae Biulgol Reservoir | 36.56528 | 127.0189 | AAK M-2382 |
| KX431629 |  | A3 | Ch15 | South Korea |  | Gae Biulgol Reservoir | 36.56528 | 127.0189 | AAK M-2382 |
| KX431600 |  | A3 | Ch17 | Russia (Asian) | Kamchatka Area | Pool near Kronoskoe Lake, Kronotsky State Natural Reserve | 54.89719 | 160.276835 | AAK M-2651 |
| KX431600 |  | A3 | Ch18 | Russia (Asian) | Kamchatka Area | Pool near Kronoskoe Lake, Kronotsky State Natural Reserve | 54.89719 | 160.276835 | AAK M-2651 |
| KX431641 |  | A3 | Ch19 | Russia (Asian) | Kamchatka Area | Pool near Kronoskoe Lake, Kronotsky State Natural Reserve | 54.89719 | 160.276835 | AAK M-2651 |
| KX431600 |  | A3 | Ch20 | Russia (Asian) | Kamchatka Area | Pool near Kronoskoe Lake, Kronotsky State Natural Reserve | 54.89719 | 160.276835 | AAK M-2651 |
| KX431586 |  | A1 | Ch21 | Russia (European) | Volgograd Area | A water body in Anisovskaya Poyma near town of Saratov | 51.7 | 46.6 | AAK M-2255 |
| KX431586 |  | A1 | Ch22 | Russia (European) | Volgograd Area | A water body in Anisovskaya Poyma near town of Saratov | 51.7 | 46.6 | AAK M-2255 |
| KX431619 |  | A1 | Ch23 | Russia (European) | Volgograd Area | A water body in Anisovskaya Poyma near town of Saratov | 51.7 | 46.6 | AAK M-2255 |
| KX431586 |  | A1 | Ch24 | Russia (European) | Volgograd Area | A water body in Anisovskaya Poyma near town of Saratov | 51.7 | 46.6 | AAK M-2255 |
| KX431608 |  | A3 | Ch25 | Russia (Asian) | Yakutia Autonomous Republic | A small dam lake near Lake Malaya Chabyda | 61.92249 | 129.4182 | AAK M-1989 |
| KX431608 |  | A3 | Ch26 | Russia (Asian) | Yakutia Autonomous Republic | A small dam lake near Lake Malaya Chabyda | 61.92249 | 129.4182 | AAK M-1989 |
| KX431607 |  | A3 | Ch27 | Russia (Asian) | Yakutia Autonomous Republic | A small dam lake near Lake Malaya Chabyda | 61.92249 | 129.4182 | AAK M-1989 |
| KX431608 |  | A3 | Ch28 | Russia (Asian) | Yakutia Autonomous Republic | A small dam lake near Lake Malaya Chabyda | 61.92249 | 129.4182 | AAK M-1989 |
| KX431636 |  | A2_2 | Ch33 | Russia (Asian) | Kamchatka Area | A pool on Stolovaya Sopka, Bering Island, Komandorsky State Biosphere Reserve | 55.24896 | 166.0214 | AAK M-2573 |
| KX431636 |  | A2_2 | Ch34 | Russia (Asian) | Kamchatka Area | A pool on Stolovaya Sopka, Bering Island, Komandorsky State Biosphere Reserve | 55.24896 | 166.0214 | AAK M-2573 |
| KX431636 |  | A2_2 | Ch35 | Russia (Asian) | Kamchatka Area | A pool on Stolovaya Sopka, Bering Island, Komandorsky State Biosphere Reserve | 55.24896 | 166.0214 | AAK M-2573 |
| KX431637 |  | A2_2 | Ch36 | Russia (Asian) | Kamchatka Area | A pool on Stolovaya Sopka, Bering Island, Komandorsky State Biosphere Reserve | 55.24896 | 166.0214 | AAK M-2573 |
| KX431642 |  | A2_2 | Ch41 | Russia (Asian) | Kamchatka Area | Kronotskoe Lake near Derzhavina Island, Kronotsky State Natural Reserve | 54.82487 | 160.28341 | AAK M-2655 |
| KX431643 |  | A2_2 | Ch42 | Russia (Asian) | Kamchatka Area | Kronotskoe Lake near Derzhavina Island, Kronotsky State Natural Reserve | 54.82487 | 160.28341 | AAK M-2655 |
| KX431644 |  | A2_2 | Ch43 | Russia (Asian) | Kamchatka Area | Kronotskoe Lake near Derzhavina Island, Kronotsky State Natural Reserve | 54.82487 | 160.28341 | AAK M-2655 |
| KX431644 |  | A2_2 | Ch44 | Russia (Asian) | Kamchatka Area | Kronotskoe Lake near Derzhavina Island, Kronotsky State Natural Reserve | 54.82487 | 160.28341 | AAK M-2655 |
| KX431583 |  | A1 | Ch50 | Russia (European) | Komi Autonomous Republic | Lake Yelya-ty, River Sysola basin | 61.58333 | 50.78333 | AAK M-1613 |
| KX431584 |  | A1 | Ch52 | Russia (Asian) | Khakass Autonomous Republic | A oxbow lake of River Abakan near Izykhskiye Kopi | 53.55623 | 91.28597 | AAK M-1648 |
| KX431585 |  | A1 | Ch53 | Russia (Asian) | Khakass Autonomous Republic | A oxbow lake of River Abakan near Izykhskiye Kopi | 53.55623 | 91.28597 | AAK M-1648 |
| KX431592 |  | A2_1 | Ch54 | Russia (Asian) | Khakass Autonomous Republic | A oxbow lake of River Abakan near Izykhskiye Kopi | 53.55623 | 91.28597 | AAK M-1648 |
| KX431593 |  | A3 | Ch55 | Russia (Asian) | Khakass Autonomous Republic | A oxbow lake of River Abakan near Izykhskiye Kopi | 53.55623 | 91.28597 | AAK M-1648 |
| KX431595 |  | A3 | Ch56 | Russia (Asian) | Chukot Autonomous Area | A pond, Lawrentia | 65.56891 | -170.011 | AAK M-1678 |
| KX431595 |  | A3 | Ch57 | Russia (Asian) | Chukot Autonomous Area | A pond, Lawrentia | 65.56891 | -170.011 | AAK M-1678 |
| KX431595 |  | A3 | Ch58 | Russia (Asian) | Chukot Autonomous Area | A pond, Lawrentia | 65.56891 | -170.011 | AAK M-1678 |
| KX431595 |  | A3 | Ch59 | Russia (Asian) | Chukot Autonomous Area | A pond, Lawrentia | 65.56891 | -170.011 | AAK M-1678 |
| KX431596 |  | A3 | Ch60 | Russia (Asian) | Chukot Autonomous Area | A pond, Lawrentia | 65.56891 | -170.011 | AAK M-1679 |
|  | KX448804 | A3 | Ch61 | Russia (Asian) | Chukot Autonomous Area | A pond, Lawrentia | 65.56891 | -170.011 | AAK M-1679 |
| KX431604 |  | A3 | Ch62 | Russia (Asian) | Chukot Autonomous Area | A pond, Lawrentia | 65.56891 | -170.011 | AAK M-1679 |
| KX431597 | KX448803 | A3 | Ch64 | Russia (Asian) | Krasnoyarsk Territory | Oxbow lake 4d, a biological station Mirnoe | 62.34 | 89.1 | AAK M-2149 |
| KX431594 |  | A3 | Ch65 | Russia (Asian) | Krasnoyarsk Territory | Oxbow lake 4d, a biological station Mirnoe | 62.34 | 89.1 | AAK M-2149 |
| KX431594 |  | A3 | Ch66 | Russia (Asian) | Krasnoyarsk Territory | Oxbow lake 4d, a biological station Mirnoe | 62.34 | 89.1 | AAK M-2149 |
| KX431609 |  | A3 | Ch67 | Russia (Asian) | Krasnoyarsk Territory | Oxbow lake 4d, a biological station Mirnoe | 62.34 | 89.1 | AAK M-2149 |
| KX431610 |  | A2_1 | Ch69 | Russia (Asian) | Tuva Autonomous Republic | A small puddle, Chirgalandy | 52.59115 | 97.22483 | AAK M-2157 |
| KX431610 |  | A2_1 | Ch70 | Russia (Asian) | Tuva Autonomous Republic | A small puddle, Chirgalandy | 52.59115 | 97.22483 | AAK M-2157 |
| KX431611 |  | A2_1 | Ch73 | Russia (Asian) | Tuva Autonomous Republic | Kaha-Hol' Lake | 51.35858 | 89.45881 | AAK M-2162 |
| KX431612 |  | A1 | Ch77 | Russia (Asian) | Chelyabinsk Area | A pool at Svyatoy Veri Island, Turgoyak Lake | 55.16053 | 60.02905 | AAK M-2167 |
| KX431615 |  | A2_1 | Ch83 | Russia (European) | Nenets Autonomous Okrug, Arkhangelsk Area | A shallow lake K3, Pechora Delta | 68.31235 | 53.08115 | AAK M-2194 |
| KX431616 |  | A2_1 | Ch84 | Russia (European) | Nenets Autonomous Okrug, Arkhangelsk Area | A shallow lake K2, Pechora Delta | 68.34158 | 53.27639 | AAK M-2201 |
| KX431616 |  | A2_1 | Ch85 | Russia (European) | Nenets Autonomous Okrug, Arkhangelsk Area | A shallow lake K2, Pechora Delta | 68.34158 | 53.27639 | AAK M-2201 |
| KX431617 |  | A2_1 | Ch86 | Russia (European) | Nenets Autonomous Okrug, Arkhangelsk Area | A shallow lake K2, Pechora Delta | 68.34158 | 53.27639 | AAK M-2201 |
| KX431618 | KX448808 | A2_1 | Ch87 | Russia (European) | Nenets Autonomous Okrug, Arkhangelsk Area | A shallow lake K2, Pechora Delta | 68.34158 | 53.27639 | AAK M-2201 |
| KX431620 | KX448805 | A1 | Ch88 | Russia (European) | Volgograd Area | A water body in Anisovskaya Poyma near town of Saratov | 51.7 | 46.6 | AAK M-2255 |
| KX431621 |  | A1 | Ch89 | Russia (European) | Volgograd Area | A water body in Anisovskaya Poyma near town of Saratov | 51.7 | 46.6 | AAK M-2255 |
| KX431620 |  | A1 | Ch90 | Russia (European) | Volgograd Area | A water body in Anisovskaya Poyma near town of Saratov | 51.7 | 46.6 | AAK M-2255 |
| KX431622 |  | A1 | Ch91 | Russia (European) | Volgograd Area | A water body in Anisovskaya Poyma near town of Saratov | 51.7 | 46.6 | AAK M-2255 |
|  | KX448809 | A2 | Ch92 | South Korea |  | Purification plant near Useong | 36.47005 | 127.0862 | AAK M-2379 |
| KX431625 |  | A3 | Ch93 | South Korea |  | Purification plant near Useong | 36.47005 | 127.0862 | AAK M-2379 |
| KX431625 |  | A3 | Ch94 | South Korea |  | Purification plant near Useong | 36.47005 | 127.0862 | AAK M-2379 |
| KX431626 |  | A3 | Ch95 | South Korea |  | Purification plant near Useong | 36.47005 | 127.0862 | AAK M-2379 |
| KX431628 |  | A3 | Ch96 | South Korea |  | Paddy rice beside Yang Hwa Reservoir | 36.33217 | 127.1652 | AAK M-2383 |
|  | KX448802 | A3 | Ch99 | South Korea |  | Paddy rice beside Yang Hwa Reservoir | 36.33217 | 127.1652 | AAK M-2383 |
| KX431630 | KX448807 | A2_1 | Ch100 | Russia (European) | Karealian Autonomous Republic | Rock pool, Kirabenavalok Cape, White Sea | 65.96318 | 34.71268 | AAK M-2453 |
| KX431630 |  | A2_1 | Ch101 | Russia (European) | Karealian Autonomous Republic | Rock pool, Kirabenavalok Cape, White Sea | 65.96318 | 34.71268 | AAK M-2453 |
|  | KX448806 | A2_1 | Ch102 | Russia (European) | Karealian Autonomous Republic | Rock pool, Kirabenavalok Cape, White Sea | 65.96318 | 34.71268 | AAK M-2453 |
| KX431632 |  | A1 | Ch104 | Norway |  | Tunevannet | 59.31 | 11.09 | AAK M-2475 |
| KX431632 | KX448810 | A1 | Ch106 | Norway |  | Tunevannet | 59.31 | 11.09 | AAK M-2475 |
| KX431632 | KX448811 | A1 | Ch107 | Norway |  | Tunevannet | 59.31 | 11.09 | AAK M-2475 |
|  | KX448812 | A1 | Ch108 | Russia (European) | Yaroslavl Area | A pond in the garden, town of Myshkin | 57.79 | 38.45 | AAK M-2484 |
| KX431633 |  | A1 | Ch110 | Russia (European) | Yaroslavl Area | A pond in the garden, town of Myshkin | 57.79 | 38.45 | AAK M-2484 |
| KX431634 |  | A1 | Ch111 | Russia (European) | Yaroslavl Area | A pond in the garden, town of Myshkin | 57.79 | 38.45 | AAK M-2484 |
| KX431598 |  | A3 | Ch117 | Russia (Asian) | Primorsky Territory | A ditch neak Khanka Lake, Khankaysky State Biosphere Reservoir | 44.66797 | 132.5089 | AAK M-2525 |
| KX431635 | KX448801 | A3 | Ch119 | Russia (Asian) | Primorsky Territory | A ditch neak Khanka Lake, Khankaysky State Biosphere Reservoir | 44.66797 | 132.5089 | AAK M-2525 |
| KX431638 |  | A2_2 | Ch140 | Russia (Asian) | Kamchatka Area | A pool on Stolovaya Sopka, Bering Island, Komandorsky State Biosphere Reserve | 55.24896 | 166.0214 | AAK M-2573 |
| KX431640 |  | A2_2 | Ch142 | Russia (Asian) | Kamchatka Area | A pool on Stolovaya Sopka, Bering Island, Komandorsky State Biosphere Reserve | 55.24896 | 166.0214 | AAK M-2573 |
| KX431636 |  | A2_2 | Ch143 | Russia (Asian) | Kamchatka Area | A pool on Stolovaya Sopka, Bering Island, Komandorsky State Biosphere Reserve | 55.24896 | 166.0214 | AAK M-2573 |
| KX431599 |  | A3 | Ch144 | Russia (Asian) | Yakutia Autonomous Republic | A puddle near the road Tyube-Namtsy | 63.19648 | 129.4849 | AAK M-2592 |
| KX431600 |  | A3 | Ch157 | Russia (Asian) | Kamchatka Area | Pool near Kronoskoe Lake, Kronotsky State Natural Reserve | 54.89719 | 160.276835 | AAK M-2651 |
| KX431646 |  | A1 | Ch164 | Russia (European) | Moscow Area | A forest pond | 55.78616 | 39.76867 | AAK M-2748 |
| KX431646 |  | A1 | Ch165 | Russia (European) |  | A forest pond | 55.78616 | 39.76867 | AAK M-2748 |
| KX431646 |  | A1 | Ch167 | Russia (European) |  | A forest pond | 55.78616 | 39.76867 | AAK M-2748 |
| KX431647 |  | A1 | Ch168 | Russia (European) | Krasnodar Territory | A bay of Kuchansky Liman | 45.22897 | 37.56946 | AAK M-2832 |
| KX431648 |  | A1 | Ch169 | Russia (European) | Krasnodar Territory | A bay of Kuchansky Liman | 45.22897 | 37.56946 | AAK M-2832 |
| KX431649 |  | A1 | Ch170 | Russia (European) | Krasnodar Territory | A bay of Kuchansky Liman | 45.22897 | 37.56946 | AAK M-2832 |
| KX431649 |  | A1 | Ch171 | Russia (European) | Krasnodar Territory | A bay of Kuchansky Liman | 45.22897 | 37.56946 | AAK M-2832 |
| KX431588 |  | A1 | Ch176 | Russia (European) | Krasnodar Territory | Flooded valley of the Anapka River | 44.90963 | 37.33387 | AAK M-2830 |
| KX431588 |  | A1 | Ch178 | Russia (European) | Krasnodar Territory | Flooded valley of the Anapka River | 44.90963 | 37.33387 | AAK M-2830 |
| KX431588 |  | A1 | Ch179 | Russia (European) | Krasnodar Territory | Flooded valley of the Anapka River | 44.90963 | 37.33387 | AAK M-2830 |
| KX431650 |  | A2_1 | Ch184 | Russia (Asian) | Yamalo-Nentrs Autonomour Area | Lake 6 near Kamenny Mys | 68.47632 | 73.57502 | AAK M-2915 |
| KX431651 |  | A2_1 | Ch185 | Russia (Asian) | Yamalo-Nentrs Autonomour Area | Lake 6 near Kamenny Mys | 68.47632 | 73.57502 | AAK M-2915 |
| KX431652 |  | A2_1 | Ch187 | Russia (Asian) | Irkutsk Area | A puddle near Muromtsovka village | 53.22578 | 105.2652 | AAK M-2939 |
| KX431653 |  | A2_1 | Ch188 | Russia (Asian) | Irkutsk Area | A puddle near Muromtsovka village | 53.22578 | 105.2652 | AAK M-2939 |
| KX431631 |  | A2_1 | Ch189 | Russia (Asian) | Irkutsk Area | A puddle near Muromtsovka village | 53.22578 | 105.2652 | AAK M-2939 |
| KX431652 |  | A2_1 | Ch190 | Russia (Asian) | Irkutsk Area | A puddle near Muromtsovka village | 53.22578 | 105.2652 | AAK M-2939 |
| KX431639 |  | A2_2 | Ch198 | Russia (Asian) | Kamchatka Area | A pool on Stolovaya Sopka, Bering Island, Komandorsky State Biosphere Reserve | 55.24896 | 166.0214 | AAK M-2573 |
| KX431613 |  | A1 | Ch204 | Russia (European) | Kostroma Area | A pond in the garden of D.P. Karabanov, town of Galich | 58.38684 | 42.36164 | AAK M-2980 |
| KX431654 |  | A1 | Ch205 | Russia (European) | Kostroma Area | A pond in the garden of D.P. Karabanov, town of Galich | 58.38684 | 42.36164 | AAK M-2980 |
| KX431654 |  | A1 | Ch206 | Russia (European) | Kostroma Area | A pond in the garden of D.P. Karabanov, town of Galich | 58.38684 | 42.36164 | AAK M-2980 |
| KX431654 |  | A1 | Ch207 | Russia (European) | Kostroma Area | A pond in the garden of D.P. Karabanov, town of Galich | 58.38684 | 42.36164 | AAK M-2980 |
| KX431655 |  | A1 | Ch208 | Russia (European) | Kostroma Area | A pond, town of Galich | 58.38619 | 42.35891 | AAK M-2984 |
| KX431655 |  | A1 | Ch210 | Russia (European) | Kostroma Area | A pond, town of Galich | 58.38619 | 42.35891 | AAK M-2984 |
| KX431655 |  | A1 | Ch211 | Russia (European) | Kostroma Area | A pond, town of Galich | 58.38619 | 42.35891 | AAK M-2984 |
| KX431614 |  | A1 | Ch212 | Russia (European) | Perm' Area | Garden pond, Las'inskie Khutora ner Perm city | 58.06149 | 55.93748 | AAK M-2992 |
| KX431656 |  | A2_1 | Ch214 | Russia (European) | Perm' Area | Garden pond, Las'inskie Khutora ner Perm city | 58.06149 | 55.93748 | AAK M-2992 |
| KX431657 |  | A3 | Ch217 | Russia (Asian) | Kamchatka Area | A small lake in the caldera of Uzon volcano, Kronotsky State Natural Reserve | 54.49925 | 159.9903 | AAK M-3003 |
| KX431657 |  | A3 | Ch218 | Russia (Asian) | Kamchatka Area | A small lake in the caldera of Uzon volcano, Kronotsky State Natural Reserve | 54.49925 | 159.9903 | AAK M-3003 |
| KX431657 |  | A3 | Ch219 | Russia (Asian) | Kamchatka Area | A small lake in the caldera of Uzon volcano, Kronotsky State Natural Reserve | 54.49925 | 159.9903 | AAK M-3003 |
| KX431658 | KX448799 | A2_2 | Ch220 | Russia (Asian) | Kamchatka Area | A lake in Urochishche Siniy Dol, Kronotsky State Natural Reserve | 54.45398 | 159.8057 | AAK M-3010 |
| KX431659 |  | A2_2 | Ch221 | Russia (Asian) | Kamchatka Area | A lake in Urochishche Siniy Dol, Kronotsky State Natural Reserve | 54.45398 | 159.8057 | AAK M-3010 |
| KX431659 | KX448800 | A2_2 | Ch222 | Russia (Asian) | Kamchatka Area | A lake in Urochishche Siniy Dol, Kronotsky State Natural Reserve | 54.45398 | 159.8057 | AAK M-3010 |
| KX431660 |  | A2_2 | Ch223 | Russia (Asian) | Kamchatka Area | A lake in Urochishche Siniy Dol, Kronotsky State Natural Reserve | 54.45398 | 159.8057 | AAK M-3010 |
| KX431601 |  | A3 | Ch224 | Russia (Asian) | Kamchatka Area | A swampy area, Drevhikh Stoyanok Cape, Kronotsky State Natural Reserve | 54.34919 | 160.3473 | AAK M-3039 |
| KX431601 |  | A3 | Ch225 | Russia (Asian) | Kamchatka Area | A swampy area, Drevhikh Stoyanok Cape, Kronotsky State Natural Reserve | 54.34919 | 160.3473 | AAK M-3039 |
| KX431662 |  | A3 | Ch227 | Russia (Asian) | Kamchatka Area | A swampy area, Drevhikh Stoyanok Cape, Kronotsky State Natural Reserve | 54.34919 | 160.3473 | AAK M-3039 |
| KX431602 |  | A3 | Ch228 | Russia (Asian) | Kamchatka Area | A swampy area 2, Drevhikh Stoyanok Cape, Kronotsky State Natural Reserve | 54.37223 | 160.3881 | AAK M-3044 |
| KX431602 |  | A3 | Ch229 | Russia (Asian) | Kamchatka Area | A swampy area 2, Drevhikh Stoyanok Cape, Kronotsky State Natural Reserve | 54.37223 | 160.3881 | AAK M-3044 |
| KX431663 |  | A3 | Ch230 | Russia (Asian) | Kamchatka Area | A swampy area 2, Drevhikh Stoyanok Cape, Kronotsky State Natural Reserve | 54.37223 | 160.3881 | AAK M-3044 |
| KX431666 |  | A1 | Ch232 | Russia (European) | Kalmyk Autonomous Republic | Puddle 2 not far from Caspian Sea | 45.31078 | 47.19498 | AAK M-3073 |
| KX431590 |  | A1 | Ch233 | Russia (European) | Kalmyk Autonomous Republic | Puddle 2 not far from Caspian Sea | 45.31078 | 47.19498 | AAK M-3073 |
| KX431590 |  | A1 | Ch234 | Russia (European) | Kalmyk Autonomous Republic | Puddle 2 not far from Caspian Sea | 45.31078 | 47.19498 | AAK M-3073 |
| KX431590 |  | A1 | Ch235 | Russia (European) | Kalmyk Autonomous Republic | Puddle 2 not far from Caspian Sea | 45.31078 | 47.19498 | AAK M-3073 |
| KX431664 |  | A1 | Ch236 | Russia (European) | Kalmyk Autonomous Republic | Puddle 1 not far from Caspian Sea | 45.31078 | 47.19498 | AAK M-3072 |
| KX431665 |  | A1 | Ch237 | Russia (European) | Kalmyk Autonomous Republic | Puddle 1 not far from Caspian Sea | 45.31078 | 47.19498 | AAK M-3072 |
| KX431589 |  | A1 | Ch238 | Russia (European) | Kalmyk Autonomous Republic | Puddle 1 not far from Caspian Sea | 45.31078 | 47.19498 | AAK M-3072 |
| KX431589 |  | A1 | Ch239 | Russia (European) | Kalmyk Autonomous Republic | Puddle 1 not far from Caspian Sea | 45.31078 | 47.19498 | AAK M-3072 |
| KX431591 |  | A1 | Ch240 | Russia (European) | Moscow Area | A pond in the alpinarium of the Botanical Garden of Moscow State Unversity | 55.7068 | 37.5258 | AAK M-3099 |
| KX431591 |  | A1 | Ch241 | Russia (European) | Moscow Area | A pond in the alpinarium of the Botanical Garden of Moscow State Unversity | 55.7068 | 37.5258 | AAK M-3099 |
| KX431667 |  | A1 | Ch242 | Russia (European) | Moscow Area | A pond in the alpinarium of the Botanical Garden of Moscow State Unversity | 55.7068 | 37.5258 | AAK M-3099 |
| KX431591 |  | A1 | Ch243 | Russia (European) | Moscow Area | A pond in the alpinarium of the Botanical Garden of Moscow State Unversity | 55.7068 | 37.5258 | AAK M-3099 |
| KX431668 |  | A2_1 | Ch244 | Russia (Asian) | Altai Territory | Dzhazator River | 49.64936 | 87.94811 | AAK M-3102 |
| KX431669 |  | A2_1 | Ch247 | Russia (Asian) | Altai Territory | Dzhazator River | 49.64936 | 87.94811 | AAK M-3102 |
