# Supplementary material for: Phylogeography of the Chydorus sphaericus Group (Cladocera: Chydoridae) in the Northern Palearctic
Source: PLoS One. 2016 Dec 19;11(12):e0168711. doi: 10.1371/journal.pone.0168711 (PMC5167426; doi:10.1371/journal.pone.0168711)
Supplement: S2 Table — (DOC) [file pone.0168711.s002.doc]

**S2 Table.** List of sequences from the GenBank that were used in our study.

| **NCBI - COI** | **NCBI - ITS** | **Clade** | **Country** | **State (if available)** | **Taxon and unique ID (if present in the Genbank)** | **Reference** |
| --- | --- | --- | --- | --- | --- | --- |
| EU719120.1 |  | A1 | Germany |  | *Chydorus sphaericus* | Belyaeva & Taylor 2009 |
| EU719122.1 |  | A1 | Germany |  | *Chydorus sphaericus* | Belyaeva & Taylor 2009 |
| EU719123.1 |  | A1 | Germany |  | *Chydorus sphaericus* | Belyaeva & Taylor 2009 |
| EU719124.1 |  | A1 | Germany |  | *Chydorus sphaericus* | Belyaeva & Taylor 2009 |
| EU719125.1 |  | A1 | Finland |  | *Chydorus sphaericus* isolate 6Fin18 | Belyaeva & Taylor 2009 |
| EU719127.1 |  | A1 | Germany |  | *Chydorus sphaericus* isolate 8Ger1a4a7 | Belyaeva & Taylor 2009 |
| EU719128.1 |  | A1 | Germany |  | *Chydorus sphaericus* isolate 9Ger4a5 | Belyaeva & Taylor 2009 |
| EU719129.1 |  | A1 | Germany |  | *Chydorus sphaericus* isolate 10Ger8 | Belyaeva & Taylor 2009 |
| EU719130.1 |  | A1 | Germany |  | *Chydorus sphaericus* isolate 11Ger5 | Belyaeva & Taylor 2009 |
| EU719131.1 |  | A1 | Germany |  | *Chydorus sphaericus* isolate 12Ger1a4a8a9 | Belyaeva & Taylor 2009 |
| EU719132.1 | EU719167 | A1 | Germany |  | *Chydorus sphaericus* isolate 13Ger1a11Ice14 | Belyaeva & Taylor 2009 |
| EU719133.1 |  | A1 | Germany |  | *Chydorus sphaericus* isolate 15Ger1Rus25 | Belyaeva & Taylor 2009 |
| EU719134.1 |  | A1 | Germany |  | *Chydorus sphaericus* isolate 14Ger1 | Belyaeva & Taylor 2009 |
| EU719135.1 |  | A1 | Germany |  | *Chydorus sphaericus* isolate 16Ger8a10 | Belyaeva & Taylor 2009 |
| EU719126.1 |  | A1 | Germany |  | *Chydorus sphaericus* isolate 7Ger3 | Belyaeva & Taylor 2009 |
| EU719137.1 |  | A1_2 | Greenland |  | *Chydorus* sp. 18Gree20a21 | Belyaeva & Taylor 2009 |
| EU822324.1 |  | A1_2 | Iceland |  | *Chydorus sphaericus* isolate 19Ice17 | Belyaeva & Taylor 2009 |
| KC020624.1 |  | A1_2 | Australia (introduced) | South Australia | *Chydorus* sp. PS-2013 isolate MYPChyd001 | Sharma & Kotov 2014 |
| EU719136.1 |  | A1-A2 | Finland |  | *Chydorus sphaericus* isolate 17Fin19 | Belyaeva & Taylor 2009 |
| EU719138.1 |  | A1-A2 | Greenland |  | *Chydorus* sp. 20Gree22 | Belyaeva & Taylor 2009 |
| EU719140.1 |  | A1-A2 | Iceland |  | *Chydorus* sp. 23Ice16 | Belyaeva & Taylor 2009 |
| EU719141.1 |  | A1-A2 | Iceland |  | *Chydorus* sp. 24Ice15a16 | Belyaeva & Taylor 2009 |
| EU719148.1 |  | A1-A2 | Finland |  | *Chydorus* sp. 32Fin19 | Belyaeva & Taylor 2009 |
| EU719139.1 |  | A2 | Russia | Tomsk | *Chydorus* sp. 21Rus23 | Belyaeva & Taylor 2009 |
| EU719142.1 | EU719170 | A2 | Norway |  | *Chydorus* sp. 25Nor12 | Belyaeva & Taylor 2009 |
| EU719143.1 |  | A2 | Russia | Yamalo-Nenets Autonomous Area | *Chydorus* sp. 26Rus24 | Belyaeva & Taylor 2009 |
| EU719144.1 |  | A2 | Russia | Arkhangelsk | *Chydorus* sp. 28Rus24a26 | Belyaeva & Taylor 2009 |
| EU719145.1 |  | A2 | Norway |  | *Chydorus* sp. 27Nor13 | Belyaeva & Taylor 2009 |
| EU719146.1 |  | A2 | Norway |  | *Chydorus* sp. 30Nor13 | Belyaeva & Taylor 2009 |
| EU719147.1 |  | A2 | Norway |  | *Chydorus* sp. 31Nor14 | Belyaeva & Taylor 2009 |
| EU822325.1 |  | A2 | Russia | Khanty-Mansi Autonomous Area | *Chydorus* sp. MAB-2008 isolate 22Rus27 | Belyaeva & Taylor 2009 |
| EU822326.1 |  | A2 | Russia | Yamal | *Chydorus* sp. MAB-2008 isolate 29Rus24 | Belyaeva & Taylor 2009 |
| EU719149.1 | EU719174 | A3 | Japan |  | *Chydorus* sp. 33Jap29 | Belyaeva & Taylor 2009 |
| EU822327.1 |  | A3 | Japan |  | *Chydorus* sp. MAB-2008 isolate 34Jap30 | Belyaeva & Taylor 2009 |
| EU822328.1 |  | A3 | Japan |  | *Chydorus* sp. MAB-2008 isolate 35Jap30 | Belyaeva & Taylor 2009 |
| GU680570.1 |  | A3 | Canada | Manitoba | *Chydorus sphaericus* complex sp. A3 | iBOL Data Release |
| HQ972026.1 |  | A3 | Canada | Manitoba | *Chydorus sphaericus* complex sp. A3 | iBOL Data Release |
| HQ972023.1 |  | A3 | Canada | Manitoba | *Chydorus sphaericus* complex sp. A3 | iBOL Data Release |
| JN233865.1 |  | A3 | Canada | Manitoba | *Chydorus sphaericus* complex sp. A3 | Jeffery et al. 2011 |
| JN233866.1 |  | A3 | Canada | Manitoba | *Chydorus sphaericus* complex sp. A3 | Jeffery et al. 2011 |
| JN233870.1 |  | A3 | Canada | Manitoba | *Chydorus sphaericus* complex sp. A3 | Jeffery et al. 2011 |
| JN233871.1 |  | A3 | Canada | Manitoba | *Chydorus sphaericus* complex sp. A3 | Jeffery et al. 2011 |
| JN233872.1 |  | A3 | Canada | Manitoba | *Chydorus sphaericus* complex sp. A3 | Jeffery et al. 2011 |
| EU719153.1 | EU719176 | A4 | Canada | Yukon | *Chydorus* sp. 39Yuk36 | Belyaeva & Taylor 2009 |
| JN233863.1 |  | SP2_NA | Canada | Manitoba | *Chydorus* sp. 2 | Jeffery et al. 2011 |
| JN233864.1 |  | SP2_NA | Canada | Manitoba | *Chydorus* sp. 2 | Jeffery et al. 2011 |
| HQ972024.1 |  | SP2_NA | Canada | Manitoba | *Chydorus* sp. 2 | iBOL Data Release |
| GU680569.1 |  | B5 | Canada | Manitoba | *Chydorus sphaericus* complex sp. B5 | iBOL Data Release |
| GU680578.1 |  | B5 | Canada | Manitoba | *Chydorus sphaericus* complex sp. B5 | iBOL Data Release |
| GU680583.1 |  | B5 | Canada | Manitoba | *Chydorus sphaericus* complex sp. B5 | iBOL Data Release |
| GU689234.1 |  | B5 | Canada | Manitoba | *Chydorus sphaericus* complex sp. B5 | iBOL Data Release |
| GU689235.1 |  | B5 | Canada | Manitoba | *Chydorus sphaericus* complex sp. B5 | iBOL Data Release |
| GU689236.1 |  | B5 | Canada | Manitoba | *Chydorus sphaericus* complex sp. B5 | iBOL Data Release |
| HQ946208.1 |  | B5 | Canada | Manitoba | *Chydorus sphaericus* complex sp. B5 | iBOL Data Release |
| HQ972027.1 |  | B5 | Canada | Manitoba | *Chydorus sphaericus* complex sp. B5 | iBOL Data Release |
| JN233873.1 |  | B5 | Canada | Manitoba | *Chydorus sphaericus* complex sp. B5 | Jeffery et al. 2011 |
| JN233874.1 |  | B5 | Canada | Manitoba | *Chydorus sphaericus* complex sp. B5 | Jeffery et al. 2011 |
| JN233875.1 |  | B5 | Canada | Manitoba | *Chydorus sphaericus* complex sp. B5 | Jeffery et al. 2011 |
| JN233877.1 |  | B5 | Canada | Manitoba | *Chydorus sphaericus* complex sp. B5 | Jeffery et al. 2011 |
| JN233878.1 |  | B5 | Canada | Manitoba | *Chydorus sphaericus* complex sp. B5 | Jeffery et al. 2011 |
| JN233879.1 |  | B5 | Canada | Manitoba | *Chydorus sphaericus* complex sp. B5 | Jeffery et al. 2011 |
| JN233881.1 |  | B5 | Canada | Manitoba | *Chydorus sphaericus* complex sp. B5 | Jeffery et al. 2011 |
| JN233882.1 |  | B5 | Canada | Manitoba | *Chydorus sphaericus* complex sp. B5 | Jeffery et al. 2011 |
| JN233883.1 |  | B5 | Canada | Manitoba | *Chydorus sphaericus* complex sp. B5 | Jeffery et al. 2011 |
| JN233885.1 |  | B5 | Canada | Manitoba | *Chydorus sphaericus* complex sp. B5 | Jeffery et al. 2011 |
| JN233886.1 |  | B5 | Canada | Manitoba | *Chydorus sphaericus* complex sp. B5 | Jeffery et al. 2011 |
| JN233887.1 |  | B5 | Canada | Manitoba | *Chydorus sphaericus* complex sp. B5 | Jeffery et al. 2011 |
| JN233888.1 |  | B5 | Canada | Manitoba | *Chydorus sphaericus* complex sp. B5 | Jeffery et al. 2011 |
| JN233889.1 |  | B5 | Canada | Manitoba | *Chydorus sphaericus* complex sp. B5 | Jeffery et al. 2011 |
| JN233890.1 |  | B5 | Canada | Manitoba | *Chydorus sphaericus* complex sp. B5 | Jeffery et al. 2011 |
| JN233891.1 |  | B5 | Canada | Manitoba | *Chydorus sphaericus* complex sp. B5 | Jeffery et al. 2011 |
| JN233892.1 |  | B5 | Canada | Manitoba | *Chydorus sphaericus* complex sp. B5 | Jeffery et al. 2011 |
| JN233893.1 |  | B5 | Canada | Manitoba | *Chydorus sphaericus* complex sp. B5 | Jeffery et al. 2011 |
| JN233894.1 |  | B5 | Canada | Manitoba | *Chydorus sphaericus* complex sp. B5 | Jeffery et al. 2011 |
| JN233895.1 |  | B5 | Canada | Manitoba | *Chydorus sphaericus* complex sp. B5 | Jeffery et al. 2011 |
| JN233896.1 |  | B5 | Canada | Manitoba | *Chydorus sphaericus* complex sp. B5 | Jeffery et al. 2011 |
| JN233897.1 |  | B5 | Canada | Manitoba | *Chydorus sphaericus* complex sp. B5 | Jeffery et al. 2011 |
| EU702105.1 |  | C | Mexico | Sonora | *Chydorus* *brevilabris* voucher ZPLMX904 | Elias-Gutierrez et al. 2008 |
| EU702106.1 |  | C | Mexico | Sonora | *Chydorus* *brevilabris* voucher ZPLMX903 | Elias-Gutierrez et al. 2008 |
| EU702107.1 |  | C | Mexico | Sonora | *Chydorus* *brevilabris* voucher ZPLMX902 | Elias-Gutierrez et al. 2008 |
| EU702108.1 |  | C | Mexico | Sonora | *Chydorus* *brevilabris* voucher ZPLMX901 | Elias-Gutierrez et al. 2008 |
| EU702109.1 |  | C | Mexico | Coahuila | *Chydorus* *brevilabris* voucher ZPLMX481 | Elias-Gutierrez et al. 2008 |
| EU702110.1 |  | C | Mexico | Durango | *Chydorus* *brevilabris* voucher ZPLMX480 | Elias-Gutierrez et al. 2008 |
| EU702111.1 |  | C | Mexico | Durango | *Chydorus* *brevilabris* voucher ZPLMX479 | Elias-Gutierrez et al. 2008 |
| EU702112.1 |  | C | Mexico | Coahuila | *Chydorus* *brevilabris* voucher ZPLMX360 | Elias-Gutierrez et al. 2008 |
| EU702113.1 |  | C | Mexico | Coahuila | *Chydorus* *brevilabris* voucher ZPLMX359 | Elias-Gutierrez et al. 2008 |
| EU702114.1 |  | C | Mexico | Coahuila | *Chydorus* *brevilabris* voucher ZPLMX358 | Elias-Gutierrez et al. 2008 |
| EU702115.1 |  | C | Mexico | Coahuila | *Chydorus* *brevilabris* voucher ZPLMX357 | Elias-Gutierrez et al. 2008 |
| EU702116.1 |  | C | Mexico | Coahuila | *Chydorus* *brevilabris* voucher ZPLMX356 | Elias-Gutierrez et al. 2008 |
| EU702117.1 |  | C | Mexico | Coahuila | *Chydorus* *brevilabris* voucher ZPLMX157 | Elias-Gutierrez et al. 2008 |
| EU702118.1 |  | C | Mexico | Coahuila | *Chydorus* *brevilabris* voucher ZPLMX156 | Elias-Gutierrez et al. 2008 |
| EU719150.1 |  | C | USA |  | *Chydorus* sp. 36Ala40a41Rus28 | Belyaeva & Taylor 2009 |
| EU719151.1 |  | C | Canada | Yukon | *Chydorus* sp. 37Yuk36 | Belyaeva & Taylor 2009 |
| EU719152.1 |  | C | USA |  | *Chydorus* sp. 38Ala42a43Yuk32a33 | Belyaeva & Taylor 2009 |
| EU719154.1 | EU719186 | B5 | Canada | Yukon | *Chydorus* sp. 40Yuk34 | Belyaeva & Taylor 2009 |
|  | EU719186 | B6 | Canada | Yukon | *Chydorus* sp. 40Yuk34 | Belyaeva & Taylor 2009 |
| EU719155.1 | EU719178 | B6 | Canada | Yukon | *Chydorus* sp. 41Yuk35 | Belyaeva & Taylor 2009 |
| EU719156.1 | EU719187 | C | USA |  | *Chydorus* *brevilabris* isolate 42NY46a47 | Belyaeva & Taylor 2009 |
| EU719157.1 |  | C | USA |  | *Chydorus* *brevilabris* isolate 43NH51 | Belyaeva & Taylor 2009 |
| EU719158.1 |  | C | USA |  | *Chydorus* *brevilabris* isolate 44NY47 | Belyaeva & Taylor 2009 |
| EU719159.1 |  | C | Canada | Newfoundland | *Chydorus* *brevilabris* isolate 47Newf37 | Belyaeva & Taylor 2009 |
| EU719160.1 |  | C | USA |  | *Chydorus* *brevilabris* isolate 48Ari44 | Belyaeva & Taylor 2009 |
| EU719161.1 |  | C | USA |  | *Chydorus* *brevilabris* isolate 49NY46a48a49ON39 | Belyaeva & Taylor 2009 |
| EU719162.1 |  | C | Canada | Newfoundland | *Chydorus* *brevilabris* isolate 50Newf38 | Belyaeva & Taylor 2009 |
| EU719163.1 | EU719188 | C | USA |  | *Chydorus* *brevilabris* isolate 51Ari45 | Belyaeva & Taylor 2009 |
| EU822329.1 |  | C | USA |  | *Chydorus* *brevilabris* isolate 45NY48 | Belyaeva & Taylor 2009 |
| EU822330.1 |  | C | USA |  | *Chydorus* *brevilabris* isolate 46OK50 | Belyaeva & Taylor 2009 |
| GU680571.1 |  | C | Canada | Manitoba | *Chydorus* *brevilabris* | iBOL Data Release |
| GU680592.1 |  | C | Canada | Manitoba | *Chydorus* *brevilabris* | iBOL Data Release |
| GU680593.1 |  | C | Canada | Manitoba | *Chydorus* *brevilabris* | iBOL Data Release |
| GU680595.1 |  | C | Canada | Manitoba | *Chydorus* *brevilabris* | iBOL Data Release |
| HQ972013.1 |  | C | Canada | Manitoba | *Chydorus* *brevilabris* | iBOL Data Release |
| HQ978735.1 |  | C | USA | Massachusetts | *Chydorus* *brevilabris* | iBOL Data Release |
| HQ978736.1 |  | C | USA | Massachusetts | *Chydorus* *brevilabris* | iBOL Data Release |
| HQ978737.1 |  | C | USA | Massachusetts | *Chydorus* *brevilabris* | iBOL Data Release |
| HQ978738.1 |  | C | USA | Massachusetts | *Chydorus* *brevilabris* | iBOL Data Release |
| HQ978739.1 |  | C | USA | Massachusetts | *Chydorus* *brevilabris* | iBOL Data Release |
| JN233856.1 |  | C | Canada | Manitoba | *Chydorus* *brevilabris* | Jeffery et al., |
| KC617536.1 |  | C | Mexico | Mexico State | *Chydorus* *brevilabris* voucher HE-234 | Prosser et al. 2013 |
| KC617537.1 |  | C | Mexico | Mexico State | *Chydorus* *brevilabris* voucher HE-235 | Prosser et al. 2013 |
| DQ310642.1 |  | C | Canada? |  | *Chydorus* *brevilabris* | De Waard et al. 2006 |
| DQ889090.1 |  | C | Canada? |  | *Chydorus* *brevilabris* | Costa et al. 2007 |
| KC617531.1 |  |  | Mexico | Campeche | *Chydorus* sp. HE-198 | Prosser et al. 2013 |
| KC617534.1 |  |  | Mexico | Campeche | *Chydorus* sp. HE-281 | Prosser et al. 2013 |
| EU719119.1 |  |  | South Africa |  | *Chydorus pubescens* | Belyaeva & Taylor 2009 |
| EU702268.1 |  |  | Mexico | Sonora | *Pleuroxus varidentatus* | Elias-Gutierrez et al. 2008 |
| EU702267.1 |  |  | Mexico | Sonora | *Pleuroxus varidentatus* | Elias-Gutierrez et al. 2008 |
| EU702264.1 |  |  | Mexico | Durango | *Pleuroxus denticulatus* | Elias-Gutierrez et al. 2008 |
| EU702263.1 |  |  | Mexico | Durango | *Pleuroxus denticulatus* | Elias-Gutierrez et al. 2008 |
| HQ972009.1 |  |  | Canada | Manitoba | *Pleuroxus procurvus* | iBOL Data Release |
| GU680574.1 |  |  | Canada | Manitoba | *Pleuroxus procurvus* | iBOL Data Release |
| DQ310644.1 |  |  | Canada? |  | *Pleuroxus denticulatus* | De Waard et al. 2006 |
